# Undertaking general practice quality improvement to improve cancer screening - a thematic analysis of provider experiences

**DOI:** 10.1186/s12875-021-01581-y

**Published:** 2021-11-17

**Authors:** Steven A. Trankle, Christine Metusela, Jennifer Reath

**Affiliations:** Department General Practice, School of Medicine, Western Sydney University, Building 30.3.24 Campbelltown Campus, Locked Bag 1797, Penrith, NSW 2751 Australia

**Keywords:** General practice, Primary healthcare support, Strategies, Australia, Qualitative

## Abstract

**Background:**

Cancer is a major cause of illness and death, and its incidence and mortality can be reduced through effective screening. In order to improve below target screening rates in one region of Australia, the local Primary Health Network supported local general practices to implement a range of quality improvement initiatives.

**Methods:**

We used a qualitative approach and interviewed 18 general practice staff and five Primary Health Network staff and contractors to understand their experiences with these quality improvement initiatives.

**Results:**

In a thematic analysis, we identified four key themes related to program set-up and implementation; patient and community education and promotion; engaging patients and communities in screening; and general practice enhancement. Program roles were clear and understood, and the program received strong oversight and support. Practice staff felt supported and motivated. Information Technology was a challenge for many practices often requiring tailored assistance. Education provided by practices facilitated patient empowerment but practice staff noted difficulties engaging patients in screening. Practices were enhanced though strong leadership and teamwork and practice learning activities.

**Conclusions:**

The tailored evidence-based quality improvement initiatives were considered effective in supporting general practices to increase their cancer screening. Key facilitators reported by participants included use of Plan-Do-Study-Act cycles, enhanced data entry and audit capacity, effective recall and reminder systems and maintaining staff motivation.

**Supplementary Information:**

The online version contains supplementary material available at 10.1186/s12875-021-01581-y.

## What is known about the topic?

Despite the recognised benefits of quality improvement in general practices there have been no evaluations of programs applying a broad range of practice-based initiatives specifically to cancer screening.

## What does this paper add?

The present paper provides evidence on how implementing a tailored combination of quality improvement initiatives in general practices can rapidly improve cancer screening participation.

## Background

Cancer is the second leading cause of death globally [1] and the leading contributor to disease burden in Australia [2]. As a major cause of illness in Australia, cancer has a substantial social and economic impact on individuals, families and the community [3]. Cancer screening programs aim to reduce cancer incidence and mortality [4] and national screening programs are available in Australia to detect breast, bowel and cervical cancer in targeted population groups [5]. Screening for cervical cancer is conducted in general practices, and they inform and encourage patients to enhance participation in national breast and bowel cancer screening programs [6]. BreastScreen Australia is a joint initiative of the Australian and state and territory governments with screening conducted by accredited screening services [7]. The Australian government funded National Bowel Cancer Screening Program is based on direct mailing of faecal occult blood detection kits to those eligible for screening [8]. Several studies have shown that GP endorsement of bowel cancer screening is an effective method of increasing participation [9]. Primary care endorsement of breast and cervical cancer screening has been similarly effective in increasing participation [10].

Primary Health Networks (PHNs) are regional organisations funded by the Australian Government to improve efficiency, effectiveness and coordination of primary health services in their region [11]. These organisations support general practices to undertake quality improvement including through use of practice data [12]. Improving participation in cancer screening in the Nepean Blue Mountains population is a priority for Nepean Blue Mountains Primary Health Network (NBMPHN), which supports general practices in four local Government areas to the west of Sydney, where participation rates across Australia’s three National cancer screening programs (bowel, breast, cervical) are below New South Wales (NSW) State averages [13–16]. Cervical screening rates in this region in 2014–15 were 53.3% (compared to the State average of 56%) and breast cancer screening rate was 46.2% (compared with 51.6%), with bowel cancer screening rate 33.3% (compared with 35.1%).

### Nepean Blue Mountains primary Health network Cancer screening program

With funding from the Cancer Institute NSW, from 2016 to 2018, the NBMPHN worked with communities to raise awareness of cancer screening and with general practices to improve screening and detection of breast, cervical and bowel cancer through evidence-based strategies tailored according to individual practice needs [17]. This included training practice staff in development of screening registers and patient recall systems, improvement of data entry, and use of audits in Plan-Do-Study-Act (PDSA) cycles. The PDSA cycles provide for iterative testing of changes to improve quality of healthcare and healthcare systems [18]. Health promotional resources were also provided for patients as well as on-line clinical and referral guidelines for general practitioners (HealthPathways). An Aboriginal liaison and a community educator were commissioned by the NBMPHN to engage patients in cancer screening (Table 1).

**Table 1:** NBMPHN Cancer Screening Program Quality Improvement Priorities and Strategies in General Practices

Prioritised quality improvement areas	Supportive Strategies
Data entry and extraction	• Establish and/or clean practice cancer screening register • Establish and utilise provider reminder and patient recall system • Support Information Technology and conduct periodic clinical audits – data analysis and feedback
Education and training	• Practice staff training including consumer engagement and quality improvement coaching • Support practice nurse participation in Well Women’s Screening course • Promote PDSA (Plan, Do, Study, Act) approaches/cycles
Resources and community promotion	• Incentive payments and continuing professional development (CPD) • Establish/promote women’s health checklist • Develop Health Pathways (localised health and referral information) for cancer screening • Provide information on the local mobile breast screening service • Provide community-based liaison workers and educators • Provide educational materials for display in practices • Provide practice information packs and information at NBMPHN website

### Research aims

As part of an evaluation of the NBMPHN Cancer Screening Program (NBMPHN –CSP), we aimed to explore how general practice and PHN staff experienced the general practice based quality improvement initiatives described above. Our findings are likely to inform other preventive health initiatives undertaken in partnership with general practices. Consumer perspectives including of community based educational activities, will be reported in a forthcoming manuscript.

## Methods

### Evaluation scope and oversight

The researchers (CM, ST, JR) from Western Sydney University developed a program logic model (PLM) in consultation with the program advisory committee, and from reviewing the literature and program documents, to guide the evaluation activities and provide a comprehensive framework for future cancer screening evaluations by the NBMPHN (Additional file 1 provides a summarised PLM). A PLM provides the capacity to extensively investigate all components of a program using multiple data collection methods and re-implement the framework for future evaluations [19].

To evaluate changes to bowel, breast and cervical cancer screening rates across its region following the strategies described above, a Western Sydney University team of qualitative researchers (CM, ST, JR) were commissioned by the NBMPHN. We aimed to explore facilitators and barriers identified by participants that could inform ongoing implementation of the quality improvement strategies.

The NBMPHN established a program advisory committee consisting of program management, and academic, clinical and consumer representatives, oversee the implementation of the quality improvements and guide the evaluation team.

### Setting

The Nepean Blue Mountains region west of Sydney, comprises four local Government areas including urban and semi-rural areas which cover almost 9179 km^2^ [21]. Transport availability, distances to services especially for outlying areas, and costs are dominant issues for the region [20]. The NBM region is aging at a faster rate compared to the rest of NSW with the increase in older persons as a proportion of the population, 5.13% compared to 3.3% across NSW between 2011 and 2026 [21].

### Study design

We conducted a qualitative evaluation of the NBMPHN-CSP using semi-structured interviews aligned with the PLM. We used the COREQ criteria as a guide for reporting our research [22].

### Participant recruitment

The program advisory committee identified and recruited a purposive sample of general practitioners, practice nurses and practice managers engaging in the NBMPHN-CSP. Potential participants were contacted using a letter of invitation and information/consent form approved by an ethics committee, and participants contacted us to schedule interviews. We stopped recruitment when we approached our target of 20 general practice participants and when adequate representation by practice staff type and locality was achieved. This predictive sampling is supported by research that notes the first five or six interviews produce the majority of new information in a data set [23]. We also sampled participants who were less positive about the program by identifying those practices that were reported by PHN staff to be less well engaged with the quality improvement interventions. The researchers contacted a small number of PHN program staff directly, including an Aboriginal liaison and community educator for interview as their perspectives of the program implementation were likely to provide important insights. The PHN staff included program officers and management who were familiar with their local area and experienced in working with general practices . No participants withdrew their consent.

### Data collection and analysis

In consultation with the program advisory committee, we developed a semi-structured interview schedule aligned with the relevant indicators of the PLM. We explored participant experiences with each of the individual quality improvement strategies, and how they were implemented, as well as any facilitators and barriers encountered. Questions also included how practices were oriented to the program, the supports provided, and outcomes at a practice level. All interviews were approximately 30–40 min in duration, audio-recorded and transcribed, and conducted one-on-one by two researchers (ST, CM), either face to face in private offices or by telephone as preferred by participants. All participants were given the opportunity to review their transcripts. We piloted the first five interviews to ensure the schedule captured the required data. Three research team members (CM, ST, JR) reviewed these first interview transcripts. The interview guide underwent further minor revision during data collection, with new questions and prompts added to explore emerging areas of interest. This process was informed by ongoing analysis of each interview as it was transcribed and by input from the program advisory committee (Additional file 2).

We conducted an inductive thematic analysis to interpret the experiences and perspectives of participants with the NBMPHN-CSP. This approach allows patterns and meanings to be captured from qualitative datasets [24]. We used a reflexive and collaborative approach to coding designed to develop a richer more nuanced reading of the data [25]. Research team members (CM, ST, JR) each coded three – four of the first eight interviews to identify patterns in the transcripts. We then agreed on an initial coding frame and coded the remaining interviews and consulted together to check and refine the emerging analysis and consider any differences in interpretation. At a final workshop, the researchers (CM, ST, JR) reviewed all interviews and agreed that saturation of codes had been achieved. The final thematic structure was also agreed to clearly and comprehensively describe our analysis (Additional file 3). We used N-Vivo 11® software to help organise the interview data.

## Results

We interviewed 23 participants over a four-month period from December 2017 including general practitioners, practice nurses and managers, and program support staff from the NBMPHN. Practice staff were drawn from the four local Government areas of Nepean Blue Mountains (Table 2).

**Table 2:** Interview participants

Participant	Number	Local Government Area
NBMPHN Staff and Contractors e.g. Aboriginal liaison (designated below as PHN)	5 (males = 3)	N/A
General Practitioners (GP)	6 (male = 6)	Lithgow **(1x PN)** Hawkesbury **(2x GP, 3x PM, 1x PN)** Penrith **(2x PM, 1x PN, 3xGP)** Blue Mountains **(3x PM, 1x PN, 1x GP)**
Practice Nurses (PN)	4 (female = 4)	
Practice Managers (PM)	8 (female = 7)	
**Total Participants**	**23**	

We identified four key themes related to program set-up and implementation; patient and community education and promotion; engaging patients and communities in screening; and general practice enhancement. These themes and the related subthemes are described in Table 3 and detailed below.

**Table 3:** Thematic structure

Major theme	Subtheme
Setup and Program Implementation	• Staff, contractor and committee roles • Governance structures • Funding adequacy and disbursement • Communication strategies • Providing program information • Practice-based support • Information technology challenges • Motivation to participate
Patient and Community Education and Promotion	• General practice screening education for patients • Suggestions to promote community-based screening education • Patient empowerment
Engaging Patients and Communities in Screening	• General practice strategies in engaging patients in screening • Challenges for general practice in engaging patients in screening
Practice Enhancement	• Leadership and teamwork • Practice learning activities • Quality improvement initiatives • Program sustainability

### Setting up and implementing the Cancer screening program

Interviewees noted that most program and committee staff, and contractors, had a good understanding of their roles, and expectations were made clear. Most staff felt well supported and knew where they could seek assistance. A strong governance structure was noted with consumer and clinical representation on the program advisory committee. Staff described the program as evidence-based, and similar to other well-evidenced programs. Funding was mostly considered adequate with practice payments described as helpful and an incentive to join the program, even if not covering additional staff time. Distribution of payments to individual GPs rather than to the practice as a whole, was raised as a concern by some interviewees.

Senior management and management here were supporting enough of the program to give us the interest and attention to help it along its way...I appreciated the early meetings which helped embed the [advisory] committee and the work they were doing. PHN 4

… it was quite useful to get financial assistance because it involved time and effort from our practice nurses. GP 1

If the funding comes to the practice, I think it will be better … doctors, when they’re doing their screening - they already get paid by Medicare, or they already charge the patient. PM 2

Communications with general practices were prioritised by the NBMPHN and supported by face to face contact enabling a good understanding of individual practice needs and how to best implement improvements. Program staff at the PHN were considered accessible and supportive, providing personalised assistance. They helped practices set realistic goals and provided information including concerning data extraction.

They began at the beginning at the program, just identifying what numbers the practice had … the size of the practice, and the staff that we have … They certainly do try to personalise it … you can get a goal that’s appropriate for this practice, so that was really good. PN 1

Practice-based support also included online programs, workshops and mentoring by other staff. Practice staff described their learning and skill development and valued improvements to patient care.

… program officers going out and really engaging and understanding what the practices need and having that two-way communication, not just the one-way communication where you’re updating them with changes. PHN 5

I had to learn it first so that I could relay it onto everybody else what is happening and if I didn’t have the PHN here to help me do that, I would be stuck. PM 4

IT support has been very good, they’ve shown us lots of opportunities that we weren’t aware of, to extract data and use that to enhance our recall programs and improve the overall care to our patients. GP 2

Information Technology was a challenge for many practices often requiring tailored support. Practice software was described as inadequate and sometimes provided unreliable or inconsistent data with staff unable to determine which patients needed screening. There was no way to flag patients if they had been screened elsewhere or did not require screening. Valuable time was taken in patient consultations when software was difficult to operate, and not all GPs used computers. Poor connectivity between software programs and problems with data entry meant PHN staff sometimes had to extract data manually.

… in [practice software] there’s no ability for them to build a register. They actually have to do advanced queries, and those advanced queries spit out different results to what [clinical audit tool] spits out. PHN 5

… you have to add the PAP smear in manually but nobody knew that that was the case with mammograms, so there’s no historical data. Even if I started it today it would only be recorded from today...and it would be wildly inaccurate. PM 1

It takes 20 clicks … you have to go into a different section, set the reminders in and a GP’s consult is 15 minutes, the patient might have multiple issues. You are now taking away from the patient. PHN 5

We’ve only got two doctors that use the computers completely...that also makes it difficult for PHN because then … this has to be done manually. PM 8

Interviewees described their motivation for participating. Some noted being motivated to provide high quality patient care through better recall and screening rates. Others were motivated to role-model these activities for GP trainees. For most interviewees, financial incentives were not considered motivating.

The cancer screening recall system wasn’t running smoothly before that, so the patient was missing care of their screening. I knew that if we got the right system in place that would be good for the patient. GP 3

They [PHN] gave us information about the cancer screening rates within our region … they were all very low so that was a big enough incentive to … increase those levels. GP 6

Maintaining motivation was considered paramount and interviewees recommended regular, ongoing PHN support including practice meetings; auditing and frequent feedback. Comparing results with other practices, was seen as a powerful motivator by some practices. Others noted the importance of celebrating successes even small ones. Some interviewees suggested that without continued motivation, screening activities could decline, especially with competing priorities and lack of time to maintain the IT skills required.

It’s helpful to have PHN representation at our meetings just to remind everyone of the support that’s there. GP 4

We are a big practice, we’re a busy practice, and at the moment clinical always comes first so patient care and treatment room duties are higher up my priority list. PN 2

### Patient and community education and promotion

Participants described practices providing patient education on screening through brochures and posters in waiting rooms, practice websites, at regular health promotion days and opportunistically during consultations. Some practice staff saw promoting screening as a way to improve knowledge and attitudes, including in the wider community.

Every month we have a health promotion drive – we have mufti days, to draw attention to it. We put the posters up, we encourage, we put pop-ups on our website for patients when they’re doing their online bookings because it goes through our website and just say, “Have you had your faecal occult checked?” whatever the topic happens to be. PM 3

I think it’s just because it’s more in the GPs minds now, so they’re likely to trigger when they’re seeing a patient and have that conversation with them. PM 7

I think if the health professionals, like the doctors and nurses, are talking about screening with them [patients] they're more likely to consider screening, or it might spread culturally to their friends or family, they might talk about screening with their friends or family. PN 3

Community-based workshops and events were also reported as promoting cancer screening. However, it was noted that some population groups such as men and Aboriginal women were hard to reach and requiring tailored education strategies. This was where involving an Aboriginal voice in the program’s implementation, who could liaise with the community, was especially helpful.

… with men [for FOBT], if there was a big football game on you’d get in early to get a ticket or you’d get in early for something that you want. Now, using the same idea, we’re saying get in early to have this test - an earlier diagnosis means better treatment...PHN 3

… gathering the people [Aboriginal women] to come in, … was the really hard part. I had to build a rapport, so I mainly concentrated on trying to get that to happen. The events were easy. It was just getting the rapport, building that … they’d come but it would take a lot of chatting to them. PHN 2

… we had an aunty [Aboriginal Elder] or two aunties mostly that used to come, and they’d provided a space to be in, and then helped with the setting up of morning teas and lunches and things. So I’ve worked closely with each of those people. PHN 2

Screening education was regarded as empowering consumers and it was also encouraging for practice staff to see patients engage in cancer screening.

The last three results in some women’s files is their mammogram, their FOBT, and a cervical screening, so they seem to be doing it simultaneously, they’re like, “okay well I’m on the bandwagon I might as well get it all done now”. PN 1

### Engaging patients and communities in screening

Cancer screening became a practice priority promoted through team meetings and informal conversations. Systems were developed or improved such as practice registers, recall and reminder systems and practice data collection and audit. Practice staff noted that regular use of these systems encouraged patient awareness and participation.

You can target those people that haven’t been through and you put a warning on that patient’s file saying, “Encourage screening” and “FOBT” or whatever it might be. PN 1

We developed a policy that people will get three reminders for things, so if they’ve got a mobile, they get a text from the practice and then if nothing happens, I write to them, and then they get a phone call… [Practice manager] developed a letter saying you’re due for your cervical screening. PN 4

However, there were challenges with other clinical priorities and similarly other patient priorities. Some patients did not respond to reminders or conversations. Equipment and technological challenges were also reported by GPs and practice staff. They related difficulties accessing bowel screening kits and with communication of results, which often required manual entry into practice software. Practice staff also described the fear and anxiety around cancer screening for some patients.

Sometimes … we’ve got other things as priority and … we need to look at that first … when you’re too busy you just let it go [cancer screening] … PM 2

… with breast screening and mammography, the reports were entered as documents when we got those reports back and therefore they had no coding on them … GP 1

Just public knowledge and fear, of getting the cervical screen done … it’s only a little town that we work in and they’re worried that we might talk about what their screening process involves, or they all talk about the myths, you know of getting a cervical smear done … PN 1

### Practice enhancement

Interviewees described increased team work with several practices arranging meetings to keep their staff informed and focused on goals and to share knowledge and expertise. However, there were also examples of poor engagement within practices which affected confidence and motivation. Some practice leaders did not appear committed to the program and passed responsibility to other staff.

We have practice meetings where we all meet over a lunch time, to update them on what’s happening. So for everyone to be aware of what we want to achieve with the data extraction, they all need to know about it and why we’re doing it. GP 2

It has been really frustrating … it led to quite a few frustrations and initially it felt like, well, why would the staff bother when there’s no direction from the leadership, and it evolved and we decided, we’ll do it ourselves. PM 3

Interviewees noted that the PHN provided learning activities and responsive support throughout the program. Information was available through websites, face to face learning and through a range of resources such as screening Health Pathways and “cheat” sheets for practice staff working with IT. Practice staff described how training improved their efficiency. They became more aware of screening rates and proficient with data entry and cleansing.

I've got a very good liaison officer at the PHN so if I do have any problems I usually just write to her or give her a ring and she will steer me in the right direction. PM 8

The PHN and the Local Health District and one of our doctors have been working a lot on pathways [Health Pathways] which I think is really helpful and the doctors are finding that really useful … because otherwise you’re just sending the patients from pillar to post. PM 1

I think it helped improve her [PN] knowledge of particular programs and probably even the importance of updating the records and keeping all the data, doing a data cleanse … . PM 6

Training was perceived to build staff skills and knowledge, and staff members took on additional roles. Some practice staff felt time constrained with pressing clinical responsibilities while others recommended additional learning activities such as peer to peer workshops and enhanced training for practices and staff with poor IT literacy.

We've got a practice nurse who previously wasn’t doing much practice nurse stuff, was doing more reception work … now we've got her doing more practice nurse things, including looking at [data extraction tool], and doing the audits and extractions from there. GP 6

It [webinar training] was always on when it was unsuitable for me, plus I find it very hard to have the time to devote to just sitting down at the computer. PN 3

We still have two GPs that don't use the computer, if they had something like IT support for them the doctors would feel more comfortable to use the computer...PN 3

Practice staff spoke enthusiastically about quality improvements and increased screening rates. Plan-Do-Study-Act (PDSA) cycles were reported to support setting realistic goals and implementation of appropriate activities. Practices refined recall and reminder systems, and developed proficiency in data entry and clinical audit, and in use of data extraction and other practice software. Access to Continuing Professional Development (CPD) points was valued by many GPs interviewed, although not all were aware of this incentive.

This program really enabled those patients to be picked up who are actually dropping out of being screened and may have been dropping out because we weren’t reminding them. GP 1

I think that’s one of the most useful tools [PDSA] actually throughout the program because it did give the admin staff a better guidance, so it did tell us what to do, how to do it, when to do it kind of thing … PM 5

We were already doing screening, but we didn't have the [data extraction tool]. Or even if we did we weren't checking on our screening rates. PN 3

The PHN considered the support they were providing to general practices as crucial in sustaining improvements achieved. Practice staff expressed commitment to continue quality improvement initiatives but some also recommended ongoing PHN support to maintain focus on cancer screening. Most respondents thought data collection and analysis should be performed by the PHN.

I try to make sure that they understand how to do that next time, because it’s important for me that once I leave the program that that becomes a sustainable practice that they are able to implement themselves. PHN 5

Once they get used to it [implementing quality improvement initiatives] they [practice staff] are quite smooth, they are quite good with it, and they are still doing it. GP 5

They [PHN] run the tests, the data extraction … probably once a quarter. I'm pretty happy with it because I don't have time to have it more often than that. PN 3

## Discussion

Our findings report the experiences of those engaged in the NBMPHN Cancer Screening Program. Analysis of the interview transcripts revealed four overarching themes: setting up and implementing the Cancer Screening Program; patient and community education and promotion; engaging patients and communities in screening; and general practice enhancement. As noted by other research using multimodal quality improvement strategies in primary care [26], our qualitative research findings support the effectiveness of such activities in engaging practice staff and patients. Facilitators and barriers identified included the importance of strong oversight and governance, as well as collaborative relationships and organisational support to overcome problems with information technology and enhance the use of data. Maintaining motivation with quality improvement was also regarded as crucial. These are discussed below with reference to the literature.

Support for quality improvement in primary care can enhance uptake of evidence-based practices and improve patient care, however quality improvement can be difficult to implement and sustain [27]. Strong program oversight and direction are essential in supporting quality improvement initiatives [28, 29]. The PHN established clear governance structures for the program, including appropriate cultural and clinical representation such as an Aboriginal community member and general practitioners. This supported effective engagement with the community and general practices.

Quality improvement also requires collaboration, with trusting and respectful relationships critical for adoption of evidence-based healthcare improvements [30]. Key to improving screening rates in Nepean Blue Mountains was the tailored support provided to general practices. Consistent with other research, our evaluation noted the many competing priorities in general practices, and limitations in information technology skills [31]. General practice staff described problems with software programs and their communication with external providers such as pathology, resulting in challenges with data availability, entry and extraction. They valued PHN staff who provided individualised practice support to address these challenges. Tailored, hands on support by PHN staff who have longstanding relationships with the practice are critical for quality improvement in general practice, however, practice staff also need to engage in the support and training provided.

Meso-level organisations such as PHNs have an important role in facilitating data measurement for quality improvement, and providing incentives and professional education [28]. The PHN assisted practices to establish and refine screening registers and recall and reminder systems, conducting periodic audits, as well as IT updates and troubleshooting. The PHN also provided training in planning and implementing PDSA cycles, and directed practices to other resources such as Health Pathways for cancer screening. Where similar support has been provided for colorectal cancer screening, especially using a team-based approach, improvements have been achieved in staff engagement and practice efficiency [32]. Training and provision of ongoing access to resources will ensure the maintenance of quality improvement.

Maintaining motivation and engagement is also critical in sustaining quality improvement [33]. Quality improvement has been described as a “team sport” where collaborating team members support and motivate each other toward common goals [34]. Our participants considered regular team meetings with engaged leaders to be highly motivating however, as noted by others [30], some of our participants reported attitudinal barriers and practice leads who were not engaged in the program. They expressed frustration and noted difficulties engaging with quality improvement when this commitment and guidance was lacking. Disengagement was said to occur when there was poor team communication and collaboration, all of which affected staff confidence. Efforts to build a team commitment to quality improvement and improving communication within teams are required when implementing quality improvement.

Most of the participating general practices described a keen sense of engagement and motivation. They received financial incentives and CPD credit for their participation but, consistent with other research, most did not consider these extrinsic rewards as key motivators [35, 36]. Instead, many described the intrinsic rewards of improved skills and efficiency, progress demonstrated by benchmarking, and especially improvements in patient care through increased cancer screening activity. They also described support from the PHN as motivating and key to sustaining practice improvements achieved. Strong ongoing collaboration with and support from PHNs are essential in maintaining motivation and engagement in quality improvement [27, 29]. To sustain quality improvement initiatives, consideration needs to be given to continuing support and ongoing motivation.

### Strengths and limitations

Our in-depth qualitative evaluation with a range of stakeholders provided valuable insights that can inform implementation of other quality improvement initiatives in general practice including beyond cancer screening.

Although we interviewed staff from practices struggling with the program, a limitation of the research is that we did not interview staff from general practices that chose not to participate in the quality improvement initiatives. This may have provided further insight into barriers and needs which if addressed could enhance general practice engagement with such programs.

## Conclusions

Primary Health Network provision of a range of evidence-based, tailored, quality improvement supports was effective in supporting general practices to improve cancer screening. Key facilitators reported by participants in our research included the need for strong program oversight, continued individualised support from PHN staff, ongoing access to resources and training, team commitment through improved communication, and continuing strategies that maintain staff motivation.

## Supplementary Information

**Additional file 1: Table 1**. Summarised Program Logic Model.

**Additional file 2: Table 2**. Interview schedules.

**Additional file 3: Table 3**. Thematic Analysis.

## Data Availability

Raw data is not available for public access due to ethics requirements of privacy in place at the time of the initiation of this study. The authors declare that de-identified data supporting the findings of this study are available within the article and an additional file. Raw data are however available from the authors upon reasonable request and with permission of Nepean Blue Mountains Primary Health Network.

## Abbreviations

CPD: Continuing Professional Development
FOBT: Faecal Occult Blood Test
GP: General Practitioner
LGA: Local Government Area
NBMPHN: Nepean Blue Mountains Primary Health Network
PDSA: Plan, Do, Study, Act approaches/cycles
PHN: Primary Health Network
PLM: Program Logic Model
PM: Practice Manager
PN: Practice Nurse
