# Supplementary material for: Undertaking general practice quality improvement to improve cancer screening - a thematic analysis of provider experiences
Source: BMC Fam Pract. 2021 Nov 17;22:230. doi: 10.1186/s12875-021-01581-y (PMC8597279; doi:10.1186/s12875-021-01581-y)
Supplement: Supplementary file 1 — Additional file 1: Table 1. Summarised Program Logic Model. [file 12875_2021_1581_MOESM1_ESM.docx]

Additional file A: Summarised Program Logic Model

Table 1: Summarised Program Logic Model

# Additional file B: Interview schedule

**NBMPHN Cancer Screening Programs Evaluation**

**Interview Schedule**

*A brief introduction will be provided. The purpose of the interview and requirements of participation including consent and audio recording, and confidentiality will be reiterated from information provided to the participant earlier. The participant will be given an opportunity to ask further questions and, if ready to proceed, will be given a consent form to sign.*

Table 2. Interview schedules

| Topic | Lead Question | Probe questions | Participants |
| --- | --- | --- | --- |
| PLM 1.1  Sources of funding  Funding allocated (and timed) according to program priorities | Could you please comment on the distribution of funding? | -How adequate was funding for the program?  -How well was funding allocated according to program priorities?  -How timely was funding allocated and disbursed? | NBMPHN staff |
| PLM 1.1  Payment incentives and CPD (provided and satisfaction) | What sort of payment incentives did you or other staff receive to support your engagement in the Cancer Screening programs?  Could I also ask if you engaged or any of your team in any Continuing Professional Development (CPD) opportunities? | -How helpful have these payments been for you (satisfaction/adequacy?)?  -In what ways have these  payments assisted you?  -Explain  e.g. - in supporting the  requirements for  implementing?  - in tracking and  managing the change  process?  -What were they?  -What has been your/others experience of CPD in this program?  -How helpful has CPD  been for you/others?  -explain  -how has CPD enhanced  your skills/other staff  skills? (also PLM4)  -Enhanced your  knowledge? (also PLM4) | General Practitioners |
| PLM 1.2 Management and Governance structures | Could you please comment on the set up stages of the Cancer Screening programs? | e.g.  -reference and advisory groups?  -ToR and meetings?  -organisational and management support of the program?  -Could you describe your experiences of these?  -What was your  involvement in these?  -How effective were  these strategies?  -Can you recommend any changes in terms of governance? | NBMPHN staff |
| PLM 1.3  Clear staff job descriptions | Were you clear about your role in relation to this initiative? | Could you please describe how you were oriented in this role?  -What support did you receive to fulfill your role?  -How satisfied are you with the support you received? | NBMPHN staff |
| PLM 1.4  Consumer reps on program committee and roles in implementation of strategies | Could you please describe your engagement in the NBMPHN Cancer Screening Programs? | -What was your role in planning of programs?  -What is your involvement with implementation of the programs?  -What about in terms of improving the programs?  -How well does NBMPHN consult with you?  -What opportunities do  you have for input?  -How satisfied are you with your involvement? | Consumer Reps on program |
| PLM 1.6 (Technology) | Could you please describe the IT support you received from NBMPHN? | -How has this assisted your use of:  -Reminder systems?  -Clinical Audit?  -Data extraction and the  PenCat tool?  -How satisfied are you with this support?  -What else would support you in this area?  - If you didn’t participate (in the webinar training), could you tell us why? | GPs and practice staff |
|  | Could you please describe the IT support you provided to general practices? | -How has this (TYPE) support been able to assist practices?  -Are practice staff developing competencies with IT as a result of PHN assistance?  -How?  -What IT challenges are practices presenting with? | NBMPHN Staff |
| PLM 1.7  (other resources)  *May need explaining-interviewer to have information of these on hand* | Have you used or accessed the:  -Practice Information  Packs?  -Cancer Screening  tools and other  resources on the  NBMPHN website? | -Did you find the Information Packs useful? -Of what value are the Information Packs to you and your staff? - -Explain?  -Did you find the cancer screening tools and other NBMPHN website resources helpful?  -Explain (in what way)?  -Is this the best way to provide this information?  -What other ways may  be useful?  -What other resources could support you? | GPs and practice staff |
| PLM 2.1  Communication/ media strategies developed to inform relevant stakeholders about program | What is your experience working with the NBMPHN to develop communication strategies for the program?  What is your experience in receiving information about the screening programs from NBMPHN? | -What has been your involvement in developing communication strategies?  -How satisfied are you with the consultation by NBMPHN about promotion and your level of input? | NBMPHN Staff and Consumer & GP reps on the Advisory Committee |
|  |  | -How do you regard promotion of the program?  - how was it promoted to  you?  -Information about screening registers?  -Information for mobile breast screening in your area?  -How satisfied are you with this level of information? | GPs and practice staff (and other agencies/primary health care services) |
|  |  | -Where did you get information about cancer (specify for each type) screening services that are available to you?  -What information has been provided?  -How useful is this information for you?  -How satisfied are you with the quality of the information provided (amount and clarity)? | Consumers |
| PLM 2.1  Commission General Practices to participate in clinical audit and QI in General Practice Program. | What attracted you to be involved in the Clinical Audit and QI initiatives for Cancer Screening?  *(Interviewer may need to explain and should have information of these on hand)* | -How were your needs considered and incorporated in the Clinical Audit and QI initiatives?  -What support was offered to you by the NBMPHN to participate in these initiatives?  -How useful were these initiatives for you?  -What recommendations for improvement would you have for future Clinical Audit and QI initiatives?  -What has it been like working with the PHN in this program? | GPs and practice staff |
| PLM 2.1  Training initiatives for General Practice and other support staff | Have you or your staff participated in any training related to cancer screening over the last 18 months? | -Who provided that training?  -How well were you oriented to the clinical audit and QI initiatives?  -was your role clear?  -What type of training did you participate in?  E.g.  -On clinical software  Including clinical audit?  -On cervical screening updates and the register?  -On recall and reminder systems?  -On Using PDSA (Plan, Do, Study, Act) approaches/cycles  **Ask for each of above**  -Was it relevant  -in what way?  -Was it useful?  -How?  -Can you comment on the “Well Women Screening Course” for PNs?  -how helpful was that  course for PNs?  -What about training for engaging men in bowel screening?  -How did you find the  presenter?  -Explain?  - Were you able to implement any learnings in your practice?  - How did training inform or change any processes in your practice?  -What changes do you recommend in training? | GPs and practice staff (and other agencies/primary health care services) |
| PLM 2.1  Practice Redesign | What activities were undertaken in your practice to achieve change?  Did you require support from the NBMPHN achieve the change? | -Who is the person most responsible for coordinating the approach in your practice?  -Did they achieve the outcome you expected?  - In what way?  -How often was support provided?  -What support was provided?  -How adequate was this support for you?  -Was it useful?  - How was it working  for you?  - is further support  needed?  -How satisfied are you with the support given? | GPs and practice staff |
| PLM 3.1  Staff/contractor satisfaction with orientation and support | Could you tell me what it was like when you started working with NBMPHN on this program? | -Was your role clear?  -How well did you understand the KPIs in your contract?  -How well were you oriented to your role?  -What support was given for your role?  -specific training? | -Aboriginal Liaison  -Bowel screening educator |
| PLM 3.1  Program implemented according to plan | Could you please describe your experience with the “implementation” of the program? | -How satisfied are you with the implementation of program initiatives (*identify each element e.g. developing a practice register / lists of patients who have and have not been screened and ask…)?*  - Were you aware of  this?  -Was it implemented in  your Practice?  -What was it like  implementing the  initiatives?  -How did it work from  your perspective (what  changed)?  -Did the activities  achieve their intended  outcomes?  -What did you need to  do to achieve those  outcomes?  -Did you/ staff  understand why they  were making changes?  -How did you engage  your staff in making  these changes?  -Any recommendations  for the future? | GPs and practice staff (and other agencies/primary health care services) |
|  |  | -What screening test(s) did you attend or complete?  -When did you last attend or complete test(s)?  -What made it easy for  you to attend or complete?  -Were there any  difficulties attending or completing?  -What was it like engaging in the *(type)* cancer screening test?  -What information and  education did you receive  about the screening test?  -Where did these  come from and *(for*  *each)* how helpful  were they?  -Did you receive any reminders about *(type)* cancer screening?  -Where did these come  from?  -How helpful were they *(for each one*)?  -What role did your GP have in supporting you to receive (type) cancer screening?  -Did your GP follow up  after screening?  -How satisfied are you  with your GP’s support?  -What part of the process would encourage you to participate in future cancer screening activities?  -What else would encourage you to participate in cancer screening in the future? | Consumers |
|  |  | -What sort of cancer prevention education was provided to you?  -How often?  -How satisfied are you  with that education?  -Recommendations for the future? | Consumers and consumer groups (e.g. men’s shed, community oriented, workplace) |
| PLM 3.2  Evaluation of program  *(although placed here according to PLM, in the interview this question should be at the end)* | This interview is part of the program evaluation – what other experience have you had of evaluation of this program?  *(Maybe outline other evaluation if they are not aware)* | -Do you think there is sufficient focus on evaluation of this program? | stakeholders GPs and Practice staff, Aboriginal liaison and PHN Educator |
| PLM 4.1  GPs /Engaged consumers and other stakeholders are informed about screening and services | Could you please describe your awareness of screening services and pathways? | -In what ways has your knowledge increased as a result of your involvement in the program?  -What has been most helpful?  - How well are you able to apply this knowledge? | GPs and practice staff (and other agencies/primary health care services) |
|  | Could you describe where you can find information on (TYPE) cancer screening programs? | -Have you been able to find the information you need?  -How helpful has that information been to you?  -Explain?  -Recommendations for other information?  -What means of receiving this information (GPs)? | Consumers and GPs |
|  | What have you learnt about cancer prevention and screening services as a result of attending education sessions (if attended)? | -Explain?  -How will this knowledge help you in the future? | Consumers |
|  | How (well) have you been able to reduce your risk of *(type)* cancer? | -How confident are you about what you can do to reduce your risk of (type) cancer?  -What steps have you taken or do you need to take? | Consumers |
| PLM 4.2  Improved consumer access and use of screening services  PLM 4.3  Program achieves stated outcomes | Could you please comment on how *(name of targeted patient groups)* are using screening services? | -What improvements have you seen in use of services?  -What are the challenges you see for different population groups to access cancer screening (e.g. CALD, Aboriginal women, and males for bowel cancer screening-note to specific LGAs)?  -What improvement have you seen in access to screening services?  -How likely are you to promote cancer screening to target population groups (CALD, Aboriginal women, and males for Bowel - note specific to LGA areas)?  -Why/why not? | GPs and practice staff |
|  | Could I please ask you about how you use *(type)* cancer screening services? | -What cancer screening services do you use?  Why/ why not?  -Has your use of these services changed because of encouragement by your GP?  - In what way?  Or encouragement in your local community?  -In what way (e.g.  Men’s Sheds,  community workshops with educators, other fora?  -What difficulties do you have in getting the cancer screening services you need?  -How satisfied are you with your access to services?  -with your use of services?  -How likely are you to use cancer screening services in the future?  - Why/why not? | Consumers (targeted e.g. Aboriginal women, men’s groups) |
|  | How do you think this program has improved the health of your patients? | -how have the cancer screening activities helped them (e.g. awareness)?  -have you noticed any changes they are making in their screening behaviours?  -or in their lifestyles? | GPs and practice staff |
|  | How do you think this program has improved your health? | -how have the cancer screening educational workshops helped you?  -have you made any changes to your lifestyle because of the program?  -explain? | Consumers |
|  | Please describe how you are working with other providers (e.g. other GPs, BreastScreen NSW, colonoscopy or colposcopy services) as a result of the NBMPHN Cancer Screening programs? | -How has your networking improved?  -What about the pathways for referral to other providers? | GPs and practice staff |
|  | I am also wondering about how your practice is working together with its team members | - Has participation in this program assisted with improved teamwork?  -How?  -Did the program lead to changes across the team?  -How?  - How are changes communicated to other team members? | GPs and practice staff |
|  | How have you developed professionally from your involvement in the screening programs | -Improved knowledge?  -Improved skills?  -Improvements in your day to day work? | GPs and practice staff |
|  | How has your practice engagement with cancer screening improved? | -Improved documentation processes?  -Improved recall/ reminder systems?  -Improved ability to audit?  -Improved identification of “who” to screen? | GPs and practice staff |
|  | How has the practice and staff capacity and knowledge improved to implement QI activities/initiatives? | -How confident is practice staff with implementation of QI initiatives?  -Of what value are the QI initiatives to practice staff?  - to patients?  -to your practice as a  whole?  - Are you planning other QI in your practice?  -What are they? | GPs and practice staff |
|  | Thinking about the program more broadly… | Were there any unexpected outcomes as a result of implementing these initiatives?  -What were they?  -How did they impact? | GPs and practice staff |
|  | Could you please describe your overall experience with the NBMPHN Cancer screening programs? | -Do you have any views about the program?  -What feedback could  you provide? | GPs and practice staff |
| PLM 5.2  Integrated and coordinated health services | Can you describe changes you have observed in terms of services working more closely together? | -How has this changed as a result of the screening program initiatives? | GPs and practice staff (NBMPHN staff) |

**Final questions:**

Is there anything we might have missed or something you would like to add?

***Participants will be thanked for their contribution.***

# Additional file C: NBMPHN Cancer Screening Program Evaluation - Thematic Analysis

The following analysis of the interview data is structured within four main categories: Setup and Program implementation; Patient and community education and promotion; Engaging patients and communities in screening; and Practice enhancement. The key themes corresponding to these categories are provided in the table below. In the analysis, each of these key themes is further elaborated by subthemes which are then described and illustrated by selected quotes.

Table 3. Thematic Analysis

| Setup and Program Implementation | Patient and Community Education and Promotion | Engaging Patients and Communities in Screening | Practice Enhancement |
| --- | --- | --- | --- |
| Staff, contractor and committee roles | General practice screening education for patients | General practice strategies in engaging patients in screening | Leadership and teamwork |
| Governance structures | Suggestions to promote community-based screening education | Challenges for general practice in engaging patients in screening | Practice learning activities |
| Funding adequacy and disbursement | Patient empowerment |  | Quality improvement initiatives |
| Communication strategies |  |  | Program sustainability |
| Providing program information |  |  |  |
| Practice-based support |  |  |  |
| Information technology challenges |  |  |  |
| Motivation to participate |  |  |  |

All participants’ voices have been presented in the analysis. Colour coding is provided to identify the stakeholder group and type of participant. The following key provides relevant colour codes and the participant acronyms.

**Participant Key**

PHN - PHN including contracted staff

GP - General Practitioners

PN - Practice Nurses

PM - Practice Managers

Consumer - breast

Consumer - bowel

|  | **Setup and Program Implementation** |
| --- | --- |
| **NBMPHN staff, contractor and committee roles**   - *Clear role and work expectation* - *Felt oriented into role* - *Felt supported in role* - *Orientation did not cover all aspects of role* - *Lack of role clarity* - *Could have benefited from a discussion about the role* - *Lack of support in role* - *Staff without a clinical background wanted better clinical understanding of the program* - *Could have benefited from better communication between meetings*   **Changes in staff challenging:**   - *Poor continuity* - *Lapses in practice visits and support* - *Slowed momentum* - *Created confusion* - *Challenges with QI activities as a result* | *I was very, very heavily involved in the development and design and then the implementation of the program activities so I felt clear from all of that work what was expected of me or what needed to be pursued or followed up PHN 4  *…it was very clear, and it was verbalised and set in writing, as well. PHN 2  *…the contract has the brief in it of what to do, what was needed, the reasons why we were doing this, basically to encourage men to use the testing kits because the figures were so low in those areas. PHN 3  *I felt that I was well oriented because I’ve done quality improvement before. PHN 5  *I felt very well supported by the organisation in that I was able to receive feedback and advice or critical review of our program and design development. And on that implementation. I had some very good relationships with my manager as well, in overseeing the programme so I felt extremely well supported in that sense. PHN 4  *I think our manager is a very good support and a lead that I have always depended on. Whenever there are any issues or updates or concerns for any of the practices we have weekly catch ups where I can debrief with her and she’s got a very, very good listening ear. She actively listens very well. She takes the information and she troubleshoots any issues, any problems, any concerns, so I feel very well supported from that side of things. PHN 5  *I think it’s been a case of if I need something then there’s people I go to. There’s been that sort of support and it’s been pretty good. PHN 3  *I would have liked the time to orient myself around all of the different components of cervical screening, mammograms, and FOBTs. I had to hit the ground running, pretty much. PHN 5  *Well, we got documents, pages of terms, questions and answers, terms and conditions, but for somebody actually sitting down and saying, look, this is what we expect from [named role] no, there was nothing like that…PHN 1  *I’m still floundering quite a bit on what the actual role was that I had there, and I probably still don’t really know. I think if a [named role] comes along, someone take the time, just sit down and say, well this is what we’re doing…I enjoyed my involvement in there, I just would have liked to have been able to contribute a little bit more knowing what I was contributing. PHN 1  *Clinically I would have liked to have a little bit more of a clinical support. PHN 5  *I’ve been involved where I can. It’s mainly academics and I’m certainly not an academic. I’ve been swatted down a couple of times when I’ve said different things…PHN 1  *Probably an email or two just to say look, following on from the last meeting, this is what we’ve done and that - I think that would help, rather than just getting, just getting in the minutes, and that’s what was discussed and then at the next meeting we talk about what happened and perhaps if there were updates in between the meetings would help the [named role]. PHN 1  *It took us I think, another five months to find another person to fill that role. So given that timeframe I think the program, implementation, I think has still very much continued but it was a challenge at the time PHN 4  *The only thing I thought that, didn’t put off the program, but put me off slightly, there was a high turnover of people. So you just got used to somebody and it was working, and it was working well, and then they’d move on, and somebody else would be coming. So you sort of felt like you had to start again. PHN 2  *…there was a period of time we didn’t do anything and no-one ring up or anything…because I think they also changed from one person to another person. PM 2  *I think the only downfall was that they kept changing people that were coming, so it was not the same person all the time. We had three or so different people that have taken over the program…I think it just got confusing as to where we were up to and then when the next person would come in and say something completely different to the one before.. PM 7  *…the handover wasn’t very streamlined. As a QI activity, we would document all of the change ideas in PDSAs. That wasn’t done…I felt it takes time to actually go through and filter through the information. What is the change idea? What are they trying to actually do? What are they trying to achieve? Have they achieved it? Has it actually been done? What’s the carry forward idea? Has that been done? PHN 5 |
| **Governance structures**   - *Program planning was evidence-based* - *Liaising with other staff in similar roles* - *Creating balance between meetings* *and planning* - *Supported by senior management in setting up governance* - *Culturally inclusive* - *Difficult getting GPs to participate in Advisory Committee* - *Setting up committees took time* - *Advisory Committee could have benefited from a review* - *Advisory Committee could have had more representation from consumers* | *I had quite a long time, roughly, a six month period to develop a whole broad structure of foundations of what the program would set out to achieve and in that time, I was able to do a lot of investigation work, whether it be accessing and reviewing the cancer screen literature, target groups that we were trying to reach with our activities. Or on the strategies to quality improve cancer screening within the general practice setting. So what had been found to be effective through the literature was what we took on PHN 4.  *…we brought a lot of those findings to involve in our efforts. I guess, I also, was fortunate to have the time to reach out to other program coordinators or officers like myself in the role and other primary health networks…PHN 4  *I think we’ve set up systems that we needed to get the job done…the balance between meetings and planning and working out what to do next has been really good, it’s been really quite well balanced with getting onto running the sessions, and then finding out from evaluations of those sessions how they went, which is where we’re up to now. PHN 3  *…senior management, and management here were supporting enough of the program to give us the interest and attention to help it along its way. So I definitely appreciated that especially at the early meetings was to help get to embed the [advisory] committee and the work they were doing and the communication with the committee members, to have that support there. PHN 4  *Just to sit in and add an Aboriginal voice to the whole thing really…PHN 2  *…probably the people that we found hardest to reach or engage initially were our clinicians, so we did have two GPs that eventually agreed to take part in the committee. But we weren't able to find them quickly or easily through our initial invitations…eventually we actually asked one or a few GPs directly if they would consider participating PHN 4  *…there was more work involved than I had initially expected, getting the terms of references together…all that documentation and the approval process was longer than I expected. PHN 4  *I think the structure of the committee would have warranted a review so two GPs representing did leave towards the end of last year, as did one of the consumer representatives left maybe mid-way through last year. And we also, had the practice manager, leave towards the end of the last year all for different and I think personal reasons. I think possibly some committee members or representatives were more active than others, so it would have been worth us reviewing the structure and make-up of the committee membership or governance. PHN 4  *…we probably could have done more to get more a voice from our consumers in the region. We probably did have a top heavy representation from either clinicians or from people working in quite familiar and confident in top-tier services, or from academia, even, they tend to take a certain tone and that may not always welcome their input or the feedback from people who are experiencing healthcare on the ground, like consumers PHN 4  *I was at the meetings, but a lot of the meeting was very, very, I found very academic and medical type stuff, and where your learned professors on the committee sort of had a lot to say and the GP lady and that sort of thing. PHN 1 |
| **Funding adequacy and disbursement**   - *Adequate funding for PHN to deliver activities* - *External grant enabled community engagement work to be funded* - *Incentive payments attract practices* - *Reimbursement for time is appreciated* - *Money helpful for education* - *Not much remuneration but anything helps* - *Costs not covered* - *Recommendation that incentive payments go to practice staff* | *…from our end I felt as though the funding was suitable or appropriate for the activities…I think it was appropriate for what we were attempting to achieve. PHN 4  *We were fortunate to receive a reasonably substantial funding grant from the Cancer Institute New South Wales for part of work which was to support the delivery of quality improvement initiative with general practices in that region. That boosted the capacity of our team to deliver activities within the cancer screening program. PHN 4  *…definitely the funding does attract people because at the end of the day without the funding we can’t really do much about it. And we’re already very limited with manpower and all that. So if there's the funding, that means we might be able to do a bit more manpower thing and then we can do more. PM 2  *Yeah, definitely a determining factor [incentive payments] and the time I’ve got on the phone now, I’ve had to basically block out half an hour where I could be seeing three patients, and that all costs time and money, so to have some sort of compensation for that, is appreciated. Whether or not we actually make any money out of it, at the end of the day with it, or it’s just been revenue neutral, I haven’t done the numbers there, I don’t know, it’s not an issue to me, but it is certainly appreciated that we are being paid. GP 2  *I think general practice usually involves doing a lot of things for which there is no monetary reward and it was quite useful to go and get financial assistance because it involved time and effort from our practice nurses. Predominantly the practice nurses and a little bit of education for the doctors as well…recompensed GP 1  *…there’s not a great deal that you get but whatever you get is a bonus. PM 4  *We just incorporate it into the everyday stuff. We didn’t particularly use it to fund anything. We just enhanced what we’re already doing in the practice. PM 7  *Even last manager, she actually end up putting a girl on just to do it - doing the whole thing - and that cost us a lot of money. PM 2  *So with the outcome payments, we only pay doctors. What about the nurses that do cervical screening tests? Just because they don’t have an item number, a provider number, we don’t pay them for those incentivised payments. That’s not right. What about the nurses spent hours calling and going back into history and putting all those recall systems? I know it’s part of their day to day, but this is not. This is an extra thing and so I say to them, look, I’m going to ask for an invoice from your practice and how you allocate that money is up to your prerogative. PHN 5  *If the funding comes to the practice, I think, will be better, I think…doctors, when they’re doing their screening things - they already get paid by the Medicare, or they already charge the patient. PM 2 |
| **Communication strategies**   - *Planning the program communication strategy* - *Establishing relationships through face to face communication* - *Tailoring communication strategies to suit needs of the practices and consumers* - *Two way communication with practices* - *Communicating using a variety of approaches* - *Being accessible, approachable and available* - *Communication to whole primary care team important* - *Helping to make personalised goals* | *We did seek to develop the communication strategy for the program with the input from marketing and communications team managers. PHN 4  *We do a lot of face to face interactions, interviews with the practices and that’s been really, really beneficial in helping us establish relationships and engagement and re-engaging especially, because [name] being absent, leaving… PHN 5  *So previously there wasn't a set system that we had in communicating to our primary care work force where and when the visiting van would be located and when. So initially we developed a strategy and this was to communicate that through our practice newsletter. We received feedback from at least a couple of GPs, that it would be far best to take a different approach. From then, I think, we actually either sent out or we made available on a website or both, posters that could be displayed with general practices that they could use to pin up in their waiting rooms. And actually let the patients, themselves, see, visually where and when the next breast screen van would be visiting. PHN 4  *…with a QI program there’s a lot of benchmarking that needs to happen and a lot of support that needs to happen, and that can only be done through the program development officers or the program officers going out and really engaging and understanding what the practices need and having that two way communication, not just the one way communication where you’re updating them with changes. You can’t just expect them to give improvement if you  haven’t given them the tools and resources to actually improve…PHN 5  *I spoke with [name] on the phone, he came and talked at one of our clinical meetings and I think I also attended one of the network nurses meetings at the PHN, so I got the information from several fronts. PN 2  *I didn’t even have to ask the question half the time, there was always the offer. If you need this or you need that or whatever you need, just tell us and there were some times where I actually took that up, and it happened immediately. They were very good. PHN 2  *They’ve been very accessible, and in the areas that they didn’t have answers for they endeavoured to get back to us with an answer…so it’s been a good collaborative team effort. GP 2  *They are very approachable. They explain things very well, the importance of it especially because I'm a clerk so you tend not to be up in the front as what nurses and doctors and that are so they’ve explained all the things to me which has been helpful. I have found them very, very good, very patient, knowledgeable…PM 8  *They’ve offered me all kinds of support. They were very helpful throughout the whole process. They were available and patient. I’ve had many rescheduling and cancellation of appointments and they supported throughout the whole process. PM 5  *I probably sound like a broken record but I couldn’t speak highly enough of them…my one for the screening I couldn’t speak highly enough of her and I don’t know her so I have only just met her. PM 4  *So they engage us by they send somebody out, and they do a print up and talk about progress on how well we're doing in those screenings…they have somebody to come out and talk to us about the importance of screening and making plans on how to improve our screening progress. PN 3  *They began at the beginning of the program just identifying what numbers the practice had and they just try and encourage it and set realistic goals that are appropriate for the size of the practice and the staff that we have…they certainly do try to personalise it, but in just setting achievable goals…and they just reinforced, it’s an individual practice, goal setting scheme, just to improve our own process so the PHN was really supportive in, it doesn’t matter what the other practices are doing, you can get a goal that’s appropriate for this practice. PN 1 |
| **Providing program information**   - *Information packs helpful, particularly for practice managers and nurses* - *Written information helpful* - *Information easy to use and understand* - *Not all staff have access to or use the PHN information* - *Information packs have limited value* - *Visits preferred by some* - *More information would have been preferred* - *Other sources used to obtain information* | *I had questions about it and had some information sent out to me from PHN, the whole information booklet that came out, so they were really good at getting me information. PN 2  *Specifically for cancer screening I guess, because we had the transitioning to the new way of doing it. We got a pack with leaflets and it was all included, so we knew what was available…We got the breast screen information for the breast screen van again; we got all that information and when I requested a couple of extra posters nothing was ever an issue. PN 2  *So, certainly with people who administered the scheme like practice manager and nurses, they [information packs] were very, very helpful because they provided a lot of resources. GP 1  *Yeah, they [information packs] were useful. It gave us an idea of how to do some of those searches. GP 6  *The information packs [were] about making sure that the results were entered correctly into our software – the information pack was really good for that, to identify the problem that we were having there…PN 1  *Most of the information that we get coming through for any of these programs has been really helpful for the guys getting it running here. PM 6  *I think the only thing really we used from the stuff that we did get from them is just the how to sheets they did for our actual clinical software, to hand out to the GPs and our practice nurse and how to record the data so it’s actually picking it up in PEN CAT. PM 7  *They gave lots of leaflets on how to extract data and use that process PN 1  *They send out a newsletter every week, so I always go through that and pick up stuff that I need from it, keep up to date with what’s going on…PN 4  *…they [information packs] were straightforward, easy to use. PM 3  *To be honest I don't know. I'd have to ask my practice manager whether she gets them [information pack], because to be honest I don't get them. PN 3  *I have a recollection of receiving a pack. In terms of using the pack, I personally haven’t used those resources…because it’s a large practice often the other point of contact is with the practice manager, but then often in relation to those services you will use our practice nurses for any promotional material. GP 4  *limited value [of the information packs], obviously verbal stuff tends to work better in this environment we find. PM 5  *…they [PHN] come and visit me and that’s enough information and that’s fine for me. PN 1  *…they [PHN] don't offer any handouts or written materials…I guess I didn't think about that they could be offering more handouts. PN 3  *So because of the changes I've had to get the information about the cervical screening actually from pathology. PN 3  *To receive information from that, I actually have to go to the breast screening place and get information, like brochures and things from them. PN 3 |
| **Practice-based support**   - *PHN is an important facilitator* - *Time is required to support skill development and knowledge* - *Visits are valued* - *Face to face visits beneficial* - *Practical support with use of software, data extraction, and clinical audit* - *PHN staff spending time with practice staff according to the needs of the practice* - *Support available on call* - *Basic IT support needed* - *Some practices did not need IT support* - *Lack of regular IT support*   **Training to set up program:**   - *Online meetings* - *Workshops* - *Learning passed on to other staff* | *…the PHN are coming out and saying well, you know, how are things going? They're giving us that little push along and reminders to say how are things going? GP 6  *…some practices really didn't have any kind of functioning reminder or recall system or knowledge of where in their clinical database their cancer screening results need to be saved or recorded in order to create an aggregate search in order to identify patients across the whole of the practice…So for those practices I often went in and held their hand and then spent sometimes an hour or hour and a half per visit, maybe for two or three visits in a row each week in order to support their skill development and knowledge and understanding of how to really use their clinical system optimally or to create an accurate and sustainable recording of screen results. PHN 4  *…we had a very good personal support. This would not have worked if they said go on-site to a website, read about it and then do it, you needed the people coming in. GP 1  *I like the face-to-face contact with the staff training because they can sit down and I can actually see which is a lot easier than sometimes seeing it on paper and that. PM 8  *The people who have come and are looking after this have been very helpful. They talk to us about some of the basic initiatives. But they also do a lot of work with our practice nurses, in terms of going into PEN CAT and drawing out those patients and making the recalls - the practical part of things. GP 6  *A representative from the PHN came out and talked me through using the PEN CAT tool, the extraction tool… she sat with me to get me to get the data with her guidance. PM 3  *Because I didn’t know my way around everything, they showed me how to use the CAT Tool. They came out and did like a training session on the Cat Tool and is it Top Bar? I am not fully trained in the Top Bar but one of the girls are coming out to do the PEN CAT and the Top Bar, just to update me on how to use it properly again. PM 4  *[Name from PHN] came out when it was first started. He would come out on roughly a monthly basis, he would check the audits to make sure that the numbers were obviously increasing, that I was doing it correctly and any help that we needed he would provide for us to try and make things a bit smoother or to maybe be able to get out and reach out to more patients. PM 8  The IT support has been very good, and they’ve shown us lots of opportunities that we weren’t aware of, to extract data and to use that to enhance our recall programs and to improve the overall care to our patients. GP 2  *[name from PHN] showed me how to put the results on the audit for the PEN CAT with the mammograms and the FOBTs. PM 8  *The staff have been very helpful in helping us to learn about the clinical audit tool - the PEN CAT tool - and how to do extractions and so forth. GP 6  *…there were practice staff that had worked in a practice for a considerable number of years and were very experienced in utilising not just one but a number of their clinical systems…so my IT support for them was more about discussing how they might extend their existing reminder system or skillset to the other two cancer screen programs with our bowel and breast cancer screening. PHN 4  *…really happy because any time we have any problems all we had to do was ring them and if they could come out straight away they would or they would send us emails to help us. PM 8  *Any information that we wanted that we couldn’t find, they’d find for us. They’re keen whenever we need help they’re happy to come out and all that. PM 7  *Basic IT support - connecting computers and things. When you had the old divisions they used to have an IT person on the staff that would go out to all the practices. They don’t do that anymore. It’s all about data extraction now, which is not really relevant for us because I can do all of that…PM 1  *Well, I’ve been using all of those things for years so I don’t know that we actually need IT support. The kind of IT support that most practices need is not available anywhere…PM 1  *We didn’t actually really need too much from them. We use PEN CAT all the time here so we were pretty familiar already with the software and how to use it…so they’d just come in and get the data off us. PM 7  *The IT support I had of the program was how do I put it exactly, it was quite good but it wasn’t well maintained. So they set up a few things here and there, and then they left us, they left us alone with it. So we didn’t get a regular IT support, which was the biggest problem. PM 5  *It was mainly set-ups, setting up the practice. I was given a little bit of training. PM 5  *I did an online meeting with them about reminders…I basically knew about the recalls and reminders. I think there was some detail in it that I thought was a bit of overkill like stuff they were doing which we actually didn’t do. It was all right, though. It gave me the basics. PN 4  *They offered that and gave me that training. And then I got to sit in on the workshops, because we always started with those ladies coming and talking about the screening and the bus and how things were, and then cancer in its stages. So I got all of it really. PHN 2  *I had to learn it first so that I could relay it onto everybody else what is happening and if I didn’t have the PHN here to help me do that, I would be stuck. PM 4 |
| **IT challenges and issues**   - *Practice software has limited capacity* - *Setting reminders can be time consuming (not intuitive) and can cut into patient consultation time* - *PHN engaging with software provider to help improve efficiency for practice* - *Data collection, recall and use must result in improved patient outcomes* - *Not all GPs use computers* - *Additional burden on PHN in extracting data* - *Practices face an information overload* - *National register and My Health record not yet fully operational* - *Need for a streamlined electronic system* | *There are no pre-existing software, so in Best Practice and Medical Director there’s no ability for them to build a register. They actually have to do advanced queries, and those advanced queries spit out different results to what PEN CAT spits out. There’s discrepancy within itself. PHN 5  *…if a patient opts out from cervical screening test because they’ve done it at another practice, or they going to have a specialist, or they just don’t want one, Medical Director doesn’t have that option. PHN 5  *There’s an issue with the software itself setting – if a result is normal or low risk for cervical screening, and the doctor has already made their decision, has looked at the results, and they have made a decision that this is a normal result, it should really prompt them that - the reminder. It doesn’t do that. It takes 20 clicks later that you have to go into a different section, set the reminders in and if you think of a consult, a GPs consult is 15 minutes, the patient might have multiple issues. You are now taking away from the patient. PHN 5  *Some of the work of our team has gone beyond what we initially set out to do…We've actually sought to engage one of the major clinical software centre’s Medical Director and there's quite a bit of work that's just starting now in terms of the Medical Director team have agreed to improve a number of the functions within their clinical software to…streamline the work flow for GPs in not only recording screen results but identify which patients might actually be ineligible or who  wish to opt out of cancer screen. PHN 4  *…obviously you need good data, but then what happens to that data afterwards? Where’s the benefit to the patient…it doesn’t seem to me that getting all this data actually has the outcome of improved patient care and there’s no point us doing it unless that’s the goal and that that actually happens. So there’s no reason for the practice to pay me however many hours to be involved in a project unless the end result is an improvement for their patients. So there seems to be a disconnect between getting the information and getting an outcome. PM 1  *We’ve only got two doctors that use the computers completely. We’ve got one doctor that uses the computer half for things and we’ve got two doctors that don’t use the computer so because we’re a real hybrid system, that’s what makes it a bit difficult. I think that also makes it a bit difficult for PHN because then they’ve got to say, well, okay then this has got to be done manually, it’s not going across properly, automatically because the doctors don’t use the computer, so they’ve had a bit of a challenge with us as well. PM 8  *I think it’s just that there are a lot of new systems that are currently – a lot of electronic based systems and it’s information overload for the practices and teaching them their software, PEN CAT, the national cervical screening, the MBS Item lists as well as My Health Record. It’s really overwhelming for them. Needs to be more streamlined. PHN 5  *At the moment, the portal that’s on the [national register] website is only a pathology portal. For me to find out this information, it’s like pulling teeth. At the end it was Telstra Health that’s managing the platform, it wasn’t even the Department of Health, Department of Human Services that was managing this. It was Telstra and the guy came back to me and said, “Oh, sorry, these people are not pathology companies. We are yet to create a medical centre portal.” PHN 5  *…every patient I think from March is now going to have a My Health Record. So, patients should actually go in and see when they have done their last cervical screening test was done, their last mammogram was done, FOBT, that does not exist. Currently it’s just a health share summary. PHN 5  *I think we need to go back to basics. There needs to be some kind of interaction between either PHNs or local health networks or somebody and either PEN CAT or the other practice management software systems so that the [screening] data is actually available because it’s not available. People think it is but it’s not. PM 1 |
| **Motivation to participate**   - *GPs noted improving patient care a motivator to participate in program* - *Aiming for excellence in care* - *Motivated to improve screening recall* - *Motivated by low rates of screening in the local area* - *Motivated to educate registrars about screening* - *Motivated to improve data quality* - *Motivated to improve practice policy* - *For some, incentive payments were a main motivator* - *Others considered these payments as token only and not motivators*   **Maintaining motivation:**   - *Information and follow up visits from PHN helped maintain motivation* - *PHN support at meetings helpful* - *Continued visits would help enable practice improvements* - *Program information through nurses meetings* - *Seeing improvements through audits* - *Statistical feedback and data comparisons help motivate and enable practice improvements* - *Celebrating successes and ensuring continuous motivation* - *Benchmarking against other practices* - *Keenness of PHN a motivator*   **Difficulties in maintaining motivation:**   - *Competing priorities*   *Staff are time poor* | *Importantly, good for the patient’s care, that’s what we’re all aiming for. So I’ll support any other program which is designed for general practice improvement, I will basically, I will support that, and I’ll fully contribute to that. GP 3  *I personally was interested in doing this research in case if I can help my patient and improve the outcome of the treatment for that. GP 5  *We’re always interested in improving the health care of our patients. So whether it’s cancer screening, or whether it’s diabetes care, or chronic disease, heart disease, whatever it might be, we've always been involved with something or other. GP 6  *…every time they [PHN] call me almost invariably we say yes, mostly just to get data and get information and even if it’s of no relevance to us particularly we still participate in a lot of things just for the greater good…PM 1  *…we’re a practice that prides ourselves on providing both a relevant and comprehensive and appropriate medical service to the community. So, we want to achieve a certain degree of excellence in that regard and therefore I think that’s another motivation. GP 4  *We probably do it more for improving the health care and improving the screening within our practice and in the area. GP 6  * it was because the cancer screening recall system wasn’t running smoothly before that, so there was some missing, the patient was missing care of their screening. I knew that if we got the right system in place, that would be good for the patient. The main attraction was basically just the patient’s care. GP 3  *…the attraction was that, because I have missed a lot of my patients, I really, really need to have this type of screening program. I personally could not track down every patient you know which patient is eligible for screening, which one is not. GP 5  *…they [PHN] gave us the general information about the cancer screening rates within our region. Obviously they were all very low so that was a big enough incentive to say well if that is low, then we should be getting on board to try and increase those levels. GP 6  *I need extra work like a hole in the head, but if they’re going to provide quality preventative medicine – general practice is all about prevention, then you just can’t stay comfortable in your own little world, you have to take on a new challenge and do a bit of extra work and reap the benefits from that. GP 2  *…because we are a training practice, we do have registrars and that was a trigger for me and the other doctors here teaching them about the benefits of screening and the benefits of setting up a system. GP 1  *Knowledge is power and if you know where you’re not doing as well as you should, that gives you some clear guidance and direction as to where you need to improve. So we had no idea about what our numbers were like until you dig down to the data and extract it and have a look at it, and yeah, so I was curious. That’s what attracted me. GP 2  *…we always think these programs are good value to our practice in terms of data quality and that sort of thing. I guess it’s why we put our hand up every time there’s one that comes out. PM 6  *…anything that can help us to achieve some standardisation of policy particularly when it comes to a screening and recall system is really quite helpful. GP 4  *Probably the incentive payments. Truthfully I have learnt here, when you have worked here, that it is all about money. PM 4  *They’re some financial support which is insignificant, that’s not any reason I would do it. It’s a couple of thousand dollars probably which probably covers our costs, but that’s not actually an incentive to do it or not. PM 1  *…they're not adequate, but it doesn’t make that much of a difference to us. We probably do it more for improving the health care and improving the screening within our practice and in the area, rather than for the money itself. GP 6  *Probably most motivated by the person who was coming from the Nepean Division of General Practice to show to them how to create a letter, how to send it and how to chase each patient, and whether they have done it or not. GP 5  *…their [PHN] support is wonderful, and definitely the funding is wonderful and then also them keep giving us the feedback and follow up checking on the program. I think it’s a wonderful technique. We have to give our progress, then our people just tend to forget and just move on and never done it anymore; you know. So they had to follow up I think to each practice. PM 2  *It was like motivating you, reading [information packs from PHN] and the benefit of the screening. GP 5  *…it’s helpful to have PHN representation at our meetings just to remind everyone of the support that’s there, but certainly there was some specific information in relation to the screening programs. GP 4  *I would like it [PHN visits] to be more frequent so then we know where is our percentage, where is our goal, how much work do we need to do to achieve that? We love achieving things. PM 2  *…they [PHN] bring it up in the nursing meetings that we have, so when all the practice nurses all get together they do a talk on that. I find it helpful…So it's [the program] being promoted through those meetings…PN 3  *Definitely the audit reports are probably the thing that urges us on the most, in terms of trying to make more and more improvements as time goes on. If we can see some of the improvements that are happening over time, then obviously that urges us to keep going and improve it further. GP 6  *I think in relation to the Faecal Occult blood testing or bowel cancer screening and then also the mammography, the receiving statistical feedback can be a good motivator just in terms of seeing what the participation rate in our practice is…and how it compares with other practices and how it compares with the national standards, I think that’s quite a good motivator. We probably don’t see those statistics very often so, you know, in some ways, you know, it may be helpful to be motivated by having some statistical feedback more often. GP 4  *They [practice staff] need continuous motivation in order for them to actually have that celebration of success…even if it’s 0.1% of an increase in a mammogram - that is still an improvement. PHN 5  *If you’re not constantly engaging them in quality improvement and showing them how their current activities is actually making or enhancing their practices, what their day to day tasks are, they’ll just forget about it. It will just go into the, you know, “I’ll do that later”. PHN 5  *…one practice that has done that, and they have an outstanding improvement in their mammograms, well, they are an exemplar for me. And I praise them and I use them as an example, so I benchmark my practices…I do create that competition between the practices. So I enable them to, well, somebody else is doing it. Give it a try. This practice has done it and they’ve received these results. PHN 5  *…they [PHN] are very keen too, the keenness, sort of rubs off on you. PM 8  *Maybe what’s lacking is motivation, and that’s where we would really probably need to do some work as in terms of motivating people to be involved in screening…I’m not particularly dissatisfied with the way things are operating, but maybe I’m not working as efficiently as I could as well. GP 4  *we are a big practice, we’re a busy practice, and at the moment clinical always comes first so patient care and treatment room duties are higher up my priority list if that makes sense…PN 2  *If you’re not always using a software people will forget how to use a software or they’re busy people…“I’ll revisit that when I have time.” PHN 5 |
|  | **Patient and Community Education and Promotion** |
| **General practice screening education for patients**   - *Education promotion through written material* - *Health promotion days to raise awareness for patients and also doctors and staff* - *Website information* - *Opportunistic screening education and preventative health* - *GPs and staff helping to change the culture of screening* - *Lack of screening education* - *Patients experiencing no screening education* | *So we get up-to-date posters, if there are any up-to-date brochures so that we can put on our front desk. We’ve got a large reception area, so we’ve got room to put pamphlets there for the patients. PM 8  *****I happened to see, just through my own visits to general practices, posters of the QI program. And they had already had posters up about the breast screening van that would be displayed when I was there. So I guess I saw the result first hand of where those posters were put up and what they look like. PHN 4  *We’ve got posters in the waiting room, so whether they’ve looked at those or there’s been more requests from patients saying, “Where’s the breast screen van at the moment?” it sort of reinforces something that they need to know. PM 3  *Every month we have a health promotion drive – we have mufti days, to draw attention to it. We put the posters up, we encourage, we put pop-ups on our website for patients when they’re doing their online bookings because it goes through our website and just say, “Have you had your faecal occult checked?” whatever the topic happens to be…It’s an initiative we’ve done anyway, but I think we did pick topics based on the involvement, of this audit. PM 3  *I can only comment from our health promotion days, like we’ve had a pap screen one, women’s health care day and information about it. There was no clinic or anything, but I guess it’s just raising awareness. PM 3  *…our practice nursing staff who are really very active in terms of promotions. So for example having almost every week of the year there’s a different sort of promotion. So, some of those promotions will relate very much to the screening programs in which case there will be posters and colour on the walls and maybe a themed day in terms of what we might wear and perhaps just in terms of raising awareness both amongst the patients, but also amongst the doctors and staff. GP 4  *When they come here, basically, we not only do their screening, we just basically for possible other related to things as well, it might be a person having another medical issue. So you get the opportunity just to talk about general health, they might be having high blood pressure or having a family history of some other type of cancer or it might be heart problem or diabetes. So, basically, it’s not only for the screening, it’s also to see the person from the general medical point of view. GP 3  *…it’s more a prevention rather than treatment. PM 5  *I think if the health professionals, like the doctors and nurses, are talking about screening with them [patients] they're more likely to consider screening, or it might spread culturally to their friends or family, they might talk about screening with their friends or family. PN 3  *I would like to think that we’ve educated a lot more 49 year olds to expect their 50 year old FOBT gift in the mail, and to get that done. PN 1  *Well, I’ve been to the doctor on a pretty regular basis, not very regular, on a regular basis. It would be useful if it was brought to your attention when you visit. Just to check even - so you have a record of, I don’t know if you get a record. Consumer - bowel 6  *****Interviewer: So what is your experience with going to your GP? Do they mention about having a breast check?  Interviewee: Mine has never. Consumer - breast 1  *…nothing has ever been raised with me, the letter that I got back saying everything’s fine and the times that I’ve gone back to my doctor he’s never said to me, “Oh, listen, that test,” blah, blah, blah, so I’m just working on the presumption that whenever anything happens, and there's a disclosure on my paperwork of who my doctor is, that that information will go to him as well. Consumer - bowel 2  *I don’t know really much about cancer, I’ve never been educated. Consumer - bowel 5 |
| **Suggestions to promote community-based screening education**   - *Word of mouth* - *Importance of elder’s and other trusted advocate endorsement of screening program* - *Opportunity for men’s groups to learn from women’s screening education* - *Identifying men’s groups and places where men meet to run education sessions e.g. workplaces* - *The internet is a main source of screening information for many patients* - *Advertise screening through public arena - schools, TV and radio* | *Word of mouth I think more than anything [to promote screening education]. Word of mouth. Consumer - breast 3  *I got some of the key aunties to come first, and say, “Look, it’s not that bad, I went, and you should come too.” So that helped very much. PHN 2  *…having people they trust [elders], just mention, “We’re going to this”…PHN 2  *…there’s less of those places for men than there are for women. It would be interesting to know about the breast cancer operations, who they’re targeting, how they’re targeting women. PHN 3  *…we’ve got to look at, well where are the men, and one of the things we’ve identified, they’re in the workplace, so we’re taking more of a stance into trying to go into workplaces, and we’re starting at the Penrith Council; hopefully we’ll be doing both clerical workers and depot workers for the Council, looking at small businesses between 50 to 100 workers. They’re often the ones that don’t get talks on any of this because they’re so small. PHN 3  *I think it would be a very worthwhile thing for those sort of things [education sessions on bowel screening for men] to be made more aware of in public arena…and that’s why I mentioned go into a police station and talk to the boss there Consumer - bowel 2  *…people started to talk about clubs and associations…they’ve given us some tips about motor cars and bike clubs, but …I think if we go to those places we’re going to find a different cohort of men from the ones who go to Men’s Sheds, and I’m guessing, going to find the men that we want who don’t know a great deal about bowel cancer or have responded not terribly well to the kits arriving. PHN 3  *There’s lots of retired groups too like Vietnam Vets, the Nepean Retired Men’s Club, and there’s the University of the Third Age…those are good places to go as well, because it’s rather spot on for our age group that we want to raise awareness around bowel cancer. PHN 3  *One of the participants did come up with a really good idea in the last session. He said, “If you want to know about groups to go and see why don’t you ask that question on the evaluation form?”…We’ve got about four or five leads from one group of 40 men that we’ll follow up. PHN 3  *…there must be heaps and heaps of walkers’ groups all over the place. And it could possibly be another avenue for [promoting bowel screening] if a little session like that was put onto the end of it. Consumer - bowel 3  *I'd recommend to someone with sufficient motivation to go online where there's an abundance of material. Consumer - bowel 7  *…a few things crop up on the Internet [about cancer screening] that I might think, oh, I’ll have a read of that Consumer - bowel 2  *To promote it [breast screening] I would put it in all the school newsletters. Consumer - breast 1  *I was able to get onto the local radio station twice, and I think we were in the paper three or four times, the local paper, as the van was coming closer. PHN 2  *I think more ads on TV which is what most people do these days, would be advantageous to point out the issues… Consumer - bowel 6  *My major influence that I can remember is that Ray Hadley was a great advocate there for bowel cancer…he was very high profile in regards to saying to people, you know, you’re best to go and talk to your doctor, it’s a simple test, to get a check for bowel cancer. He raised it as an issue and it was out there before thousands and thousands of people. Consumer - bowel 4 |
| **Patient empowerment**   - *Some patients are already proactive with screening* - *Patients motivated to seek screening information* - *Patients become proactive after receiving screening education* - *Increased patient awareness of importance of screening* - *Screening awareness brings about lifestyle changes* - *Patients engaging in screening is encouraging for GP practices* - *Patients encourage others to be screened* | *I’ve always done my breast screen. I’m nearly 75. I have never ever missed it. Well, I think because I’m not being smart, but I think I am just pro-active and involved in a lot of things. Consumer - breast 3  *I’m aware enough of the condition to take the initiative myself, I was briefed on the original diagnosis to obtain observations every two years, so I take it upon myself to organise that. Consumer - bowel 7  *I did make it my business to research my conditions online…but I think there's a lot of people that aren’t as motivated as what I am, as aware of what I am, they will benefit from being pointed to a lot of self-education and, again, I’ve said online a couple of times and I would reaffirm that. Consumer - bowel 7  *If you're not satisfied too much then have a frank discussion with your GP, or when you attend the clinics, as I do, have a discussion with the nurse, in that case maybe there's also a specialist nurse, or again they may say, have a word with Dr so and so, and so and so, and try and get some more information that way. Consumer - bowel 1  *Heightened awareness of the importance of early prevention, early intervention, and prevention of the type that we receive from [PHN educator], public education, and public awareness campaigns. Consumer - bowel 7  *I'm more aware of them now, I'm more proactive in them [bowel screening]. Consumer - bowel 1  *…it [cancer screening program] has increased their awareness, how to prevent it, what to look for it, how to change their lifestyle, how to look after themselves. GP 5  *I think it’s encouraged women to take on all three aspects of cancer screening, and men, both of them. To be just really proactive and just promoting that preventative health is most definitely better than trying to treat the cure or take a cure. PN 1  *…the last three results in some women’s files is their mammogram, their FOBT, and a cervical screening, so they seem to be doing it simultaneously, they’re like, “okay well I’m on the bandwagon I might as well get it all done now”. PN 1  *Where they’re actively trying to increase their activity, they’re maintaining good weight loss, or maintaining a healthy weight bracket, they’re asking to see dieticians and physios…I think they’re embracing that and looking after themselves a bit better. PN 1  *…encouraged when the patients come in here, telling us, “I received this letter and I’m here just to do such and such test.” GP 3  *…you're passing it on anyway, and that may help somebody else. Because you've just actually sat there and talked about the issues...Consumer - bowel 1  *And I know that I’ve encouraged a lot of people to do the test that I’ve had since I was diagnosed. And a lot of people have followed that advice. But you’ve got to be proactive, you’ve got to point it out to people. Because people just, you know, it’s flippant, it’s passing them by. It won’t happen to me. I try and persuade my son to go for tests, you know, it’s ridiculous and now he does. Consumer - bowel 6  *…we tried to get people’s names in for the breast screening. **Y**eah, trying to encourage. Consumer - breast 1 |
|  | **Engaging Patients and Communities in Screening** |
| **General practice strategies in engaging patients in screening**   - *Building practice registers* - *Data collection and audit,*   *software recall and reminder*   - *Follow up of patients that need to be recalled* - *GPs also need reminding to follow up* - *GP double checks recall* - *An escalating reminder system* - *Screening information sent by practice with reminders* - *Program raises awareness for GPs and PNs* - *Talking more with patients about screening* - *Rotating whole of practice focus on different health areas* | *We try to find under screened women…PM 3  *…if you’re practicing preventive medicine, you need to have systems in place and those systems need to be consistent and consistently used by everybody so that patients don’t drop through holes in the system GP 1  *We are quite big on our data entry and data extraction so just trying to educate the staff and the admin people of entering certain information in certain places where it gets picked up by the tools, and just making sure that there is a unified approach of doing things. PM 5  *So at least we get clean data, so when we audit it we can get a clearer picture. PN 3  *…we have reminders pop up in our management system. So when somebody’s due for a PAP smear it will pop up. PM 1  *…because the recall system is there [name of receptionist] checks the recall system every single day, to see who is for recall. GP3  *You can target those people that haven’t been through and you put a warning on that patient’s file saying, “Encourage screening” and “FOBT” or whatever it might be that’s not done and that’s where you can allay the myths and the fears and ask them why. PN 1  *I just re-ran all the reports last week to go through them again and try to get everybody up to date again…every week [name] runs the report of anyone that is on the recall list, and then we chase up. PN 4  *…it [cancer screening program] has enabled the staff now to go and establish systems of screening and follow-up, which are now being implemented and are being continued GP 1  *…making sure there is a recall and we’ve actually got people really routinely getting their screening done in a preventative health measure. PN 1  *…we’ve picked up people who needed to be re-recalled. There are some people who attend screening services regularly and religiously because they have their own system of ensuring that, but there are other people who don’t, and they’ll do it only if they’re reminded and so that depends on us to remind them. GP 1  *So one of the RNs is calling some patients who were overdue to see if they’d had it somewhere else. PM 6  *…if we didn’t have that recall where you phone patients, lot of people just forget. PM 4  *…we’d put warnings into the warning screen so that they [GPs] could see. It’s only a couple of weeks ago I said to them, “Are you guys still watching the warning screen and trying to chase people who need these things”, and “Oh, yeah, yeah, yeah”. PN 4  *I’m still working with the doctors trying to say, if that phone is flashing in the patient’s file in Medical-Director, that means there’s an out of date recall and it would be really great if you could look at that and just see if it’s still relevant. PN2  *So we pretty much just get the data, find out who has done it, who hasn’t done it. And then we either call the patient to come in to see the doctors or a lot of the time, we’re more likely to give it to the doctors first and let them check on the list. And then they will come back saying, “Yes, call this one, yes, call this one.” The practice manager will just pick up the phone, or sometimes she sends them an SMS message and just tell them, look, you’re whatever is due. Come in and make an appointment and see your doctor. PM 2  *I ring or write a letter to remind people. We developed a policy that people will get three reminders for things, so if they’ve got a mobile, they get a text from the practice and then if nothing happens, I write to them, and then they get a phone call. PN 4  *…we’ve developed letters on file to send out and the practice manager had sent a couple of - because patients were saying they wanted to know more information - so she developed a letter that when we sent it out saying you’re due for your cervical screening, there’s a lot of information in that letter for the patient to look at. I think it’s covered fairly well. I looked at the letters and gave the okay on them. PN 4  *What we found is the mailing system is more sort of effective and more accessible for the patient, and they do appreciate it rather than giving them a call. GP3  *I think it’s just because it’s more in the GPs minds now, so they’re likely to trigger when they’re seeing a patient and have that conversation with them. So they’re likely to get that prompt to actually get the service done. PM 7  *I guess just being more aware and when I have patients at my desk, I just always will check with them if they’re due for screening. I ask the doctors to check and [name] certainly does that with the PAP smears. PN 4  *Certainly, I’ve been more proactive in getting the information out there and focussing on the waiting room information and just making sure we had the information available to pass on to the doctors. PN 2  *I'm more likely to talk to patients about whether – how long it's been since they've last been screened. PN 3  *We explain what it is, why we’re doing it [bowel screen], and hopefully make him comfortable that it’s not as bad as it probably sounds. GP 6  *They’re [practice staff] pretty good at having that chat with patients and recommending them to go and have the services done when they’re in those age brackets and all that kind of stuff. PM 7  *…you get to know the patients personally so you can actually talk to the patients on that level and stuff like that if they ask and give them that bit more support as well instead of just from the doctor. PM 8  *Every month we have a topic and so we put up information sheets around the practice…And we promote that with the doctors as well to try and encourage them to identify patients and to disseminate information to them during the consults…PM 3  *…we’ve got a clinical meeting next week and I’ve said to the doctors, “We need to talk a bit more how we’re entering the cancer screening.” PN 2  ***…**it was by talking about it, it made us more aware how important it is to keep on top of screening and cancer screening. PN 3  *So when we have our clinical meetings, we try and bring these topics up GP 6 |
| **Challenges for general practices in engaging patients in screening**   - *Competing priorities* - *Time poor* - *Practice reception staff guard access to GP* - *Not all practices have cultural diversity* - *Not previously targeting different population groups at risk* - *Reluctance of patients to engage in screening* - *Screening kits not received* - *Information disconnect with pathology providers* - *Need for ease, accuracy and consistency of data entry* - *Needing to manually code mammogram reports* - *Inconsistent and unreliable data entry for mammogram* - *Non-standardised language between providers* - *Coding not consistent for faecal occult screens* | *We want to be responsible. I mean, sure sometimes we might have to say, oh, I’m so sorry, we’ve got other thing is priority and more higher and we need to look at that first. PM 2  *****…trying to pin doctors down sometimes is a bit tricky because when they’re at work they’re booked pretty solidly and free time to get together is a bit tricky. PN 2  *It’s whether they’ve got time to discuss that with you because they’re overwhelmed or and they don’t have time to sit there, like the bedside manner thing. The family doctor, sitting there, giving you all the advice under the sun doesn’t seem to exist anymore. Consumer - bowel 6  *…but the thing is when you’re busy and all that if there’s no cancer screening, when you’re too busy you just let it go, you don’t look at it or do it too much. PM 2  *When [Aboriginal liaison] took over this job she went around to all the doctors’ surgeries and seen the doctors. But some of the people would say, “Oh no, he hasn’t got time,” which they did at my doctors…and he said they had never ever told him…where the girls at the office said he didn’t have time. Consumer - breast 2  *…we’re relatively homogenous. I think most of our patients tend to get lumped into one group because they are, you know, we don’t have a massive ethnic subpopulation or a massive Aboriginal subpopulation. GP 1  *…we didn't really target any particular cultural group. So I wouldn't think there was an actual jump in one particular cultural group as opposed to another, because there wasn't any targeted promotion of cancer screening towards one particular cultural group. PM 3  *Aboriginal populations I would think would be lower but I don’t know that, but I would think it would be lower screening rates. Younger women are a bit hard to get on board. PM 1  *I know that I can tell them that there are services from the PHN that can drive them to appointments and things like that, if they need too, for Aboriginal people. PN 3  *…you have people that are external to us who, for example, under screened, disadvantaged communities like CALD groups or Aboriginal groups. They’re not really part of the QI programs and there is no target for us to be working with them but we really should be. If we’re doing a QI program well, and we’re looking at under screened patients we should be targeting those groups, working with those external organisations. So that integration hasn’t really happened from what I can see, very well. But again, those communities are difficult to engage with, so maybe a QI program is not the best way, I don’t know. I have no idea, I can’t comment on that. PHN 5  *They’re kind of reluctant, they just want to come in, take their script and walk out, so that’s the main struggle we had. PM 5  *We still have a few patients that refuse to have pap smears. As much as you talk to them, they still refuse to do it. PM 8  *…we are still waiting for some of the patients because we did send a letter and everything to them, but they have not yet done it, so we need to do more. A bit of encouraging the patient to participate. GP 5  *The hard part is convincing patients to come in…when we've got a list of say pap smear patients who have not had one for the last say four years or so, and we've called them up, and for whatever reason, they don’t come in. I think patient engagement is probably the bit that needs the biggest push. GP 6  *I rang the Department of Health and they were supposed to send me out 20 kits but to date I haven’t received them. I mean I can send out letters to each individual that it’s time for their bowel screening but it is no good if they don’t have a kit… PM 4  *…the breast screening results aren’t being delivered electronically into a codable field automatically from the BreastScreen New South Wales to the GP practice - that technology or system capability doesn't exist yet in New South Wales. PHN 4  *a similar challenge was presented for bowel screening. There was an option for the contracted pathology provider, Dorevitch pathology, to send results electronically to practices but that wasn’t the default setting. So most practices in the region are probably across the state are just receiving all of their results by letter. PHN 4  *…it's not staying within a codable field in the clinical software…There's no way to know who has or who hasn't had a mammogram. PHN 4  *…it [program] will only become reliable if this information is being fed back to PEN CAT. So there must be a way for them to find mammograms in patient records. I don’t know because I’m not the computer expert. PM 1  *Because a lot of information is picked out of the program, but if it’s not recorded in the right spot, then it won't pick it out. GP 6  *…with breast screening and mammography, the reports were entered as documents when we got those reports back and therefore they had no coding on them so in fact, we discovered from an IT point of view, that our mammograms were not well documented and so therefore we had to go through a process of actually doing it…GP 1  * The letter got scanned into the file and because it didn’t have the right descriptors that the report picked up, we actually started off looking like we had no breast screening done at all. The first few months was a big effort and we had to re-put all the results in…I think it would be good if it came through with the correct heading that will pick it up in the reports. PN 4  * the reception girls are the ones that are inputting the data and it’s not picking up the data that they’re putting in. It’s one of those that the GPs then have to manually put in once they see the letter. PM 7  *…the doctors would have to manually record every mammogram and they don’t do that, and nor should they have to really. That’s the whole point of us spending $15,000 a year on a program to do our data and it’s not consistent. If you’re relying on individual practitioners to remember to manually enter mammogram results then that’s unreliable. The fact that PEN CAT can’t pull that information out is not helping us understand how we’re going. PM 1  *…it’s been known for, like, 15 years, that you have to add the PAP smear in manually but nobody knew that that was the case with mammograms, so there’s no historical data. Even if I started it today it would only be recorded from today and trying to get everybody to start doing a new process like that, it would be wildly inaccurate. PM 1  *…part of our problem with just screening for certain words is that from this end, it’s not always identified in the same way by every pathology provider or radiology department. I think there are issues around a lack of common terms. PN 2  *We do for letters coming in, but we had to change how it was being set in the system so it would pick up that the provider, Breast Screen New South Wales, that was actually a mammogram and not just a correspondence letter. PM 6  *…the faecal occult blood screening, we also found that the different pathology laboratories coded them differently so we needed to keep an eye on how they came in and I think that involved maybe talking to a couple of the path labs and, sort of, seeing if they could modify their codings. GP 1 |
|  | **Practice Enhancement** |
| **Leadership and Teamwork**   - *Engaging staff in understanding reasons why the practice is involved in the program* - *Time is required for regular staff meetings and clinical meetings* - *Clear communication within team enables discussion of the program* - *Engaging with staff about the program has helped improve teamwork* - *Including and involving all members of the practice team* - *Staff share information and expertise with the team* - *Services are working more closely together* - *Health Pathways a facilitator* - *Integration of services in early stages* - *Service provider network remained the same*   **Disengagement:**   - *Lack of understanding reasons why practice is involved and the importance of teamwork* - *Improved communication between staff needed* - *Lack of consistent team meetings* - *More regular meetings needed* - *Lack of financial reward a disincentive to participate in program* - *Lack of commitment from GPs to follow through with program* - *Need for more involvement from GPs* - *Disengagement from leaders is frustrating for practice staff* - *Need for engaged leadership* | *…we have practice meetings where we all meet over a lunch time, just to give them an update on what’s happening. So for everyone to be aware of what we want to achieve with the data extraction, they all need to know about it and why we’re doing it. GP 2  *So we have a partner meeting regularly, once every two months with the owners of the practice and we sort of set agendas on what we want to do and then have clinical meetings on alternate months with all the doctors and nurses. So we talk about any changes and things that we want to do. We completely changed the way we do our recall systems from a paper based system to an electronic system. So all of that’s discussed regularly at clinical meetings and anything else. PM 1  *We do have a regular practice meeting where between 100% and 80% of the doctors attend on a weekly basis…but there are times where very often, all the doctors in the practice get together and we will discuss things and we discuss clinical scenarios, we discuss difficult patients, we pick each other’s brains and we also try to formulate strategies, including this one. GP 1  *…so it’s a team approach and we’ve got a clinical meeting next week and I’ve said to the doctors, “We need to talk a bit more how we’re entering the cancer screening.” PN 2  *once the girls knew that we’d signed up for this and we needed to get our recalls out there and our numbers up, we’ve all had a collaborative approach, so teamwork within the practice, has improved. GP 2  *The receptionists have given information to the female members about, like an information questionnaire thing that they give to the doctors about how long has it been since they've been screened, in order for us to improve our screening rates. PN 3  *Like the paps for the pap trained nurses...I think it’s been a bit more of, they realise they don’t have to do it all themselves. PM 7  *The nurses will ask some of the doctors who know how to use PEN CAT about how to do things. So we’ll go and help them, in terms of doing extractions or helping with recalls. I guess that improves our teamwork. GP 6  *It’s fed into our…ethos of practice. It being a teaching practice, I go and feedback and try and involve the registrars in what’s happening, and obviously I try and get the practice nurses to both teach them and try and get the registrars to use the programs which the practice nurses supervise, administer or are involved in. GP 1  *I get extra stuff which I read and then sometimes I’ll forward it on to the practice manager if there’s something that she needs to implement into - in fact, the last PHN had something about the bowel screening programs, so I sent it on to her so that she’s aware. PN 4  *…it was more talking to the doctors and providing them with information, so they could then answer their patients’ questions and just dealing with, well, what’s important? How do I deal with this change and what does this mean, and that’s where I got information from the PHN, so I understood it, because once I can get a grip on it then it’s easier to pass on to someone else. PN 2  *That started to happen in [name of place], which I believe from talk, hasn’t happened for a very long time. There’s lots of people that I spoke to about, they kept saying things like, “We’re last on the line,” and “We don’t get services,” and all of that sort of thing, but the PHN came together with the hospital staff and [name of Aboriginal Liaison] and there were lots of people that weighed in on the conversation. PHN 2  *I think as a result of our program we had very good examples of services working together…There were some good examples of the Breast Screening Assessment Service working with the community health team at the Local Health District. They offered a number of screening and information days for Aboriginal women in the region. PHN 4  *I know that the PHN and the Local Health District and one of our doctors have been working a lot on pathways [My Health Pathways] which I think is really helpful and the doctors are finding that really useful…because otherwise you’re just sending the patients from pillar to post. PM 1  *having that explicit integration with I guess, LHD specialists, from my perspective, hasn’t really happened. It’s probably in its infancy stage because of My Health Pathways but…it hasn’t really, from what I can see. PHN 5  *The services have always been around, and we use the same type of services that we've always used. So no, I wouldn’t say our network has increased any more than what it was before. GP 6  *[name of practice] for example, their reception staff are the ones doing the work, but because they’re doing all the work and the doctors are not telling them why they’re doing the work, they become disgruntled. It’s lack of communication. It’s so important to have that early on at the establishment stage, the orientation stage, really having that team effort. Yes, I am the doctor. Yes, I’m delegating this to you and I appreciate you doing this. PHN 5  *A little more communication on our side of things between different people… PN 2  *General staff I would say not very confident…and this is due to the lack of regular communication in regards to the topic. PM 5  *…add more regular meetings with the staff which providers are also included in the meetings, I think that would be the way going forward. That would create a greater interest from the providers which obviously reflects on the rest of the team, the providers are reluctant to work on the program due to financial reasons, which makes it quite limited. PM 5  *I think more input from the medical staff, which is quite hard to enforce, they’re always busy…and that means they need to be rewarded, I think, slightly higher for their time for those meetings as well. So maybe if they come down and meet the staff, the medical staff for half an hour or so, to compensate them I think that would attract a few more medical practitioners to get involved. PM 5  *We had three GPs who committed to be involved in the audit, and unfortunately the three GPs for various reasons didn’t get involved. PM 3  *I suppose, in my opinion I think if the doctors got more involved. A lot of the time they leave it up to the staff and that and I don’t think that the doctors actually get involved. PM 8  *****I think there was a little bit of a gap or a bit of a distance between the doctors involved in this activity and the PHN…I was somewhat removed from the interaction with the PHN GP 4  * It has been really frustrating…I think it led to quite a few frustrations and initially it felt like, well, why would the staff bother when there’s no direction from the leadership, and I guess it evolved and we decided, we’ll do it ourselves. PM 3  *I guess encouraging doctors, if they’re going to commit to something, they should do it, rather than say, yeah, yeah, yeah, let’s do this, and nothing happens with it…you need to be more selective on how you get to take part. PM 3 |
| **Practice learning activities**  Staff have a variety of preferences for resources:   - *Online* - *Emails* - *Cheat sheets* - *Face-to-face* - *Webinars* - *Newsletters and paper-based materials* - *PHN staff* - *Some staff preferred resources from other provider websites* - *Training of staff in using clinical software* - *Staff attend webinar training* - *Training enables staff to share knowledge with colleagues and patients* - *Training increased staff awareness of screening rates* - *Training increased staff knowledge of record keeping and data cleansing* - *Training increased staff knowledge in reminder and recall systems* - *Upskilling of staff* - *Practice staff extend their roles* - *Increased knowledge enables staff to optimise their role* - *Time is needed to devote to training*   **Suggestions:**   - *Improvement Foundation could be used to provide resources and development* - *Make use of shared communication platform* - *Peer to peer learning workshops* - *Incentivised workshops and training* - *IT support for staff with poor IT literacy* | *I prefer just looking it up online. I think it’s easier. You can find more up to date information than paper. PM 7  *I google around until I find the resources that we need. PM 3  *I've used the website. PN 3  *I guess in the first instance, emails with links are always helpful and then looking online, I love cheat sheets. So, any cheat sheet that can be given, that would be great. PM 3  *…if you need detailed information or a crib sheet for doing something over and over again, you print it off. GP 1  *I prefer face to face. But webinar if we can’t do that. PN 1  *I read my newsletters and things that come through, so I get that information that way. PN 2  *I still like paper - I’m old-fashioned in that way - for me to have it written down so I can read it and copying to share with doctors is really quite helpful, and I like having those resources so if a patient has a question I can say, here. PN 2  *I've got a very good liaison officer at the PHN so if I do have any problems I usually just write to her or give her a ring and she will steer me in the right direction. PM 8  *Sometimes we do ask the PHN. I think the nurse in particular contacts the PHN for resources. PM 3  *I probably went straight to the Cancer Council if I needed to order anything. I probably didn’t look at the PHN website as such PN 2  *I think we went straight to the websites of the actual provider as opposed to theirs [PHN]. Sometimes their information on their sites is not as accurate as the direct one. PM 7  *…the reception staff got a lot of training through that as well as just, you know, learning how to use MD better. And I suppose we all did. We all learnt to use MD a little bit more efficiently for entering coded things and making sure that results are entered properly. PN 1  *When I first started, I went to a day’s training on the clinical software, because I hadn’t used the package that they have here. PN 4  *PHN have come out and they’ve given one on one tutorials or instructions to two of the main practice staff members who have been involved in the program. GP 2  *I go to most of the sessions they have of different things, but I do do other webinars when they have webinars and they’re good. PN 4  *It [webinar training] brought me up to speed with why we were changing and to understand, just that understanding as why the screening process was changing and why they thought it was necessary to change, and then I was better able to communicate with my colleagues why it was changing or with my patients. This is why we are doing it, so it helped me talk about it better. PN 2  *So it [training] improved, I guess improved knowledge of - an awareness of how we are in our screening rates, and how we should improve our screening. PN 3  *…reception used some training aids to improve the handling of results within the practice. PN 1  *Because she learns from the people coming from the Area Health, so just like sitting there with her behind the front desk, and just like telling her what to do and she’s just been involved with the data, like setting up the stuff in the computer… GP 3  *I think it helped improve her [PN] knowledge of particular programs and probably even the importance of updating the records and keeping all the data, doing a data cleanse and that sort of thing. PM 6  *I think the girls are far more knowledgeable about how the systems works now, particularly, with the faecal occult blood recall and the mammograms. So I don’t think that my knowledge has increased any more, but it’s good to have the girls trained up, so that they understand why we’re doing it and how important it is for early detection. GP 2  *…since there’s a new system is in place, she knows more like how to get the patient on time here, and how to record the patient, how to communicate with the patient, and all this stuff basically. I see lots of improvements in basically all aspects of being at the front desk as a receptionist. GP 3  *…another thing is the general education of the staff members…we trained a few staff members… and how to, yes, just try to take a bit of load of the doctors and the others back. PM 5  *…it improved skills of my secretary, how to do this type of data collection, and notifying patients who require this type of preventative screening such as bowel cancer screening, or breast cancer screening for breast cancer. GP 5  *We've got a practice nurse who previously wasn’t doing as much practice nurse stuff, but was doing more reception work. So now we've got her to do more practice nurse things, including looking at PEN CAT, and doing the audits and extractions from there. GP 6  *…so the receptionist gives the people a bit of a nudge. Then if the patient wants to talk or ask some questions about it…we send the majority of the people down to the nurse if the doctors are busy or they’ve already seen the doctor…the nurses down there as well can talk to the patients so it has improved I think the knowledge especially with the receptionists. PM 8  *I think increased knowledge and increased awareness and they [practice nurses] are able to work more towards the top of the scope of their practice. PM 3  *Well it [webinar training] was always on when it was unsuitable for me, and plus I find that it very hard to just sit down at a computer and have the time to devote to just sitting down at the computer. PN 3  *…in hindsight, in regard to the planning stages of the project if they would have approached the Improvement Foundation to mentor and guide and provide resources and development for the project, a lot of streamlining could have happened. PHN 5  *We live in social media technology world where they expect that two-way communications to happen…I think if we set up a base camp platform for the 18 practices and one person has queries about how to get FOBT results electronically and I answer the question, well somebody else can go in there and see that QI information or be able to quickly access all of the resources, all the tools that we have set up for them, rather than emailing me and saying, “Hey, can you resend this cheat sheet in.” PHN 5  *it’s about utilising the advanced exemplar practices that are actively doing things and helping others. And showing their peers that they can help each other. And this is something that is missed out as part of this program, with the collaborative wave approach there’s learning workshops. And in these learning workshops they’re really encouraged to have that peer to peer interaction where the doctors talk to the other doctors, which they might not have the opportunity…Nurses can talk about the fact that, “Does your practice do this?” “Yeah, my practice does.” “Oh, okay.” Then I’m normal. I don’t feel victimised or disadvantaged in any way. PHN 5  *I would recommend maybe if they put a few workshops and maybe five or six training sessions at the practice compulsory of that, within the program, which would, one of them be initial set up, second is, just following up and making sure everyone is on track, and create a bit of an incentive environment so that everyone knows what they’re doing throughout the whole process…specific staff training to train people and regular follow ups, I would say. PM 5  *If they were to I guess give classes for the staff and GP's and things like that, that would help. Because we still have two GP's that don't use the computer, so if they had something like IT support for them so the doctors would feel more comfortable to use the computer, that'll be helpful. PN 3 |
| **Quality Improvement (QI) Initiatives**   - *Establishing screening registers* - *Carrying out clinical audit to identify who to screen* - *Utilising recall and reminder systems* - *Problems identified with setting appropriate recalls* - *Improvement in entry of screening results* - *More accurate audit data* - *Improved awareness of screening rate data* - *Improvement in screening rates* - *Improvement in all three areas of cancer screening* - *Need for feedback to understand improvement* - *Screening systems were already in place* - *Using PDSA cycles to encourage quality improvement* - *PDSA cycles can be time consuming and difficult with limited staff capacity* - *Opportunity for Continuing Professional Development (CPD) points are welcomed* - *CPD helps in practice quality improvement* - *Not all GPs were aware of CPD opportunities through the program* - *Practice staff are time poor* - *Need for more staff* - *Staff face competing priorities*   **Program sustainability:**   - *Commitment to continuing with quality improvement initiatives* - *Creating sustainability* - *Support needed to keep motivating practices* - *Perception that ongoing data collection responsibility lies with PHN* - *Perception that PHN is time and resource poor*   **Suggestions for future programs:**   - *Outsource quality improvement component* - *Additional staffing to resource program* - *Need highlighted for other types of screening* - *Focus on one cancer screening type at a time* | *…this program really enabled those patients to be picked up who are actually dropping out of being screened and they may have been dropping out because we weren’t reminding them… GP 1  *[screening registers] done more regularly in the practice now. GP 6  *…when you looked at our recall system we had recalls that were so out of date it was amazing, and we’ve been gradually tidying those up. PN 2  *…it [recall and reminder system] was there before, but it wasn’t that sort of like, it wasn’t running smoothly, so we now have it running in a much, much a better way. GP 3  *…the nurses have been extremely proactive adapting to the new program and continuing with appropriate recall. GP 4  *…this [cancer screening program] enabled us to set up formalised systems, which either were incomplete or we did not have. It made us formalise or tighten up our reminder system more so. Up until that stage, each individual doctor had sort of had their own way of reminding follow-up...there was the potential for reminders not to be generated as efficiently or effectively or at all. GP 1  *…it has enabled the staff now to go and establish systems of screening and follow-up, which are now being implemented and are being continued GP 1  *…now they’re being taught to document it properly so the next recall list will be a lot more accurate now, yes. PM 2  *…it can be tricky to differentiate between why a person has had their mammogram and what sort of mammogram it is and when the recall needs to be done. So, yes, I think probably in a way we’ve identified more problems than we have solved problems when it comes to developing a process for appropriate recall. GP 4  *You could see their change in completed cancer screening results over time from the base line data collection through to the three month and then six month data collection…the higher number in proportion of patients were getting screened and those results were being saved in the right place and therefore completed cancer screen results could be identified, using a search tool. Whereas that was previously invisible. PHN 4  *We’ve certainly improved from baseline by a substantial amount… but I suspect that most of that is improved collection of data rather than more people…because I don’t think we’ve improved by 5%. I think that our data collection has improved by 5%. PM 1  *…looking at the numbers in front of me now, I think that what I’ve observed is that I don’t know how many increased mammograms have been done. All I know is that the number of reported mammograms has increased. GP 2  *I don’t think there was any value added to the patients. I think, really things were happening as they always happened, we were just more aware of results. PM 3  *It helps us with showing us how many percentages that we're getting…it gives more of a general overview of whether we're improving, like one month or the numbers are going down as to how well our recall and screening procedures are going. PN 3  *We were already doing screening, but we didn't have the PEN CAT tool. Or even if we did we weren't checking on our screening rates. PN 3  *The program was a success. I think it did help improve the screening rates of our patients. PN 2  *I’m actively doing more pap smears and reminding people that come through, “Have you had your screening done?”…I think we definitely have an increase in uptake in a lot of the screening services. PN 1  *So do you mean do I think that the way we are running it now, we are getting more people in for screening? We do now because I do that recall. PM 4  *I think the only thing we’ve noticed is the increased amount of paps that we’re getting come through. PM 7  *Well, definitely with National Bowel Screening, it has definitely increased, probably quadrupled…and also we can see an increase of the result of the mammogram as well. GP 5  *I think in that regard with the pap smears and the mammograms they’re more - the patients are using them a little bit more. The bowel cancer one not 100 per cent sure because I get the letters from the patients but they normally come from the National Bowel Cancer Screening Program. PM 8  *…thanks to this new screening system. So we do, I think, improving in all the three areas, breast screening, the bowel screening, and the cervical screening, yes. GP 3  *In terms of just getting the message across, it might be useful to have some face-to-face feedback on the program…in relation to what the program has achieved and perhaps opening up a potential for discussion again within our practice as to what we have achieved. But, also probably looking at whether or not there’s been any change in the statistics, so what our participation rates are like and also encouragement to recruit. GP 4  *We already had ours set up and running through our clinical software so we just kept using that. PM 6  *We had a good recall system anyway. The nurses do the recall system so they were pretty well on top of anything like that. PM 8  *I think we’ve had a well-developed system and of course there’s a long history with the cervical screening program so that I think that actually works very efficiently in our practice. And, I’m not sure how much the PHN support would have really impacted on that. GP 4  *I think that’s one of the most useful tools [PDSA] actually throughout the program because it did give the admin staff a better guidance, so it did tell us what to do, how to do it, when to do it kind of thing, which made life a little bit easier, so that was a very good initiative. PM 5  *…educating the staff in the practice about what goals we were setting during the PDSAs, and then establishing new routines for the results part in the practice. PN 1  *I think the last time they [PHN] came out we went through some PDSAs and things so they give us ideas, or we can bounce things around with them…PM 6  *I suppose the most frustrating part for me is trying to do the PDSAs. I know they’re a really good tool for setting goals and trying to move the practice forward. But as a timeframe - I suppose I am talking from my perspective, we don’t have a practice manager, so I wear that hat as well as the practice manager, so I found that very time consuming, when I’ve got huge clinical demands. You know, just to have to be doing another piece of paper - I’ve found that sometimes really frustrating. PN 1  *It provided quality improvement and, you know, sort of, Category A CPD points which are always welcome. GP1  *…there’s another program with the [name], we use that when they are running the cancer screening for cervical cancer, bowel cancer, and breast cancer. I think that there is some CPD points there, probably would be by mid or end of 2018, I would look into this program. GP 3  *Most of the practice are involved in some sort of research or some sort of QI programs…I guess we’re doing it more for our practice perspective rather than a personal thing. But I think there are some QI points associated with it, but I'm not 100% sure. GP 6  *I haven’t actually been involved in any CPD activities specifically in relation to the program. In fact I’d have to say I wasn’t aware that they were available. GP 4  *I just could have done with some more time sometimes, but that’s a chronic problem. PN 2  *…it was a burden, because it was an extra load on what we normally do. So if they had somebody that could come out and just help us do that, that would have been better. PN 3  *…we were given some suggestions about additional recalls to be focusing on…there just wasn’t the manpower to get another recall up and running. PM 3  *Well we would prefer to have more staff to meet all the goals that they were demanding. PN 3  *If they could have provided a specific staff member to sit down and give some time to help with all the initiatives, because there were quite a lot of initiatives that they were doing. That would have been better. PN 3  *The project wasn’t really at the forefront of other people’s minds. So, there was a lot of distractions that drew our attention away from really giving it a really good go. PM 3  *We've all got the drive in us to do these things, but other things - clinical medicine gets in the way GP 6.  *we went through accreditation later last year, so that was a huge time consuming deal and then there was some post-accreditation things that needed to be sorted out, and that then took priority again, so unfortunately, I guess, for me, sometimes it’s been on the backseat because there have been other more critical issues to deal with. PN 2  *Once they get used to it [implementing QI initiatives] they [practice staff] are quite smooth, they are quite good with it, and they are still doing it. GP 5  *I think even once this project is finished, I think that we’ll still just go on doing what we’ve been doing so that we are catching up with people and just giving people the best opportunity that we can. PN 4  *I think for us it’s not something that will be stopped, it’s going to be an ongoing process. PM 6  *I try to make sure that they understand how to do that next time, because it’s important for me that once I leave the program that that becomes a sustainable practice that they are able to implement themselves. PHN 5  *…if we had someone who would come along on a semi-regular basis just to give us a bit of a nudge to say how are you going with this? What are you up to on that? And maybe to help us implement some of those things…I think that on a regular basis would keep things going for a lot longer than if a program, for instance, just stops suddenly. GP 6  *We can do these audits and we can do them for six months, or a year, or two years. But I guess the question is how do we keep going year after year, ongoing? That means that the GPs or whoever the clinical leads are, need that little bit of encouragement or a nudge just to say keep doing this kind of thing. GP 6  *If we keep getting some of these reports that are coming through, that’s the encouragement that we need to keep doing these sort of things. I just hope it doesn’t, you know, come to a stop at some stage. GP 6  *…they [PHN] run the tests, the data extraction…probably once a quarter. I'm pretty happy with it because I don't have time to have it more often than that. PN 3  *…unfortunately, I don’t think the PHN had enough manpower to be able to do it for us so we’ve got to do it ourselves…at the end of the day I think PHNs before still had limited time to be able to just focus on - because there were so many practices to do it. PM 2  *I think the project would have benefited from maybe outsourcing someone for the quality improvement part to an external organisation such as the model of the Improvement Foundation, even for them to implement like the model of improvement theory and model. Just because really, when I came from the COPD project which was outsourced to that organisation, the foundation, and the foundation to put a PDSA cycle, the theory behind it, how you make small incremental changes in a small period of time, test it out and make a change. That theory or that model wasn’t really implemented so the structure of this current program hasn’t been as well as informed…PHN 5  *If there was an external person who’s able to drive some of the quality improvement activities that currently happen, would have influenced at the earlier stages of the program how to actually implement the model of improvement, that could have had a good change on the current program. The management - making sure that there is a staff member that’s able to cater to all of the practices. PHN 5  *…but we need to do other disease screening as well, in order to improve the patient outcome and patient participation. GP 5  *I think realistically maybe focusing on one of the screening at a time. I think doing the three, threw the practice a little bit, because we were trying to focus on too much in one go. I think it if had have been done as separate ones, particularly the breast screen one maybe as a separate one as opposed to the other two. PM 7 |
